# Changes in Human Milk Fat Globule Composition Throughout Lactation: A Review

**DOI:** 10.3389/fnut.2022.835856

**Published:** 2022-05-12

**Authors:** Caroline Thum, Clare Wall, Li Day, Ignatius M. Y. Szeto, Fang Li, Yalu Yan, Matthew P. G. Barnett

**Affiliations:** ^1^AgResearch Ltd, Te Ohu Rangahau Kai, Palmerston North, New Zealand; ^2^Faculty of Medical and Health Sciences, The University of Auckland, Auckland, New Zealand; ^3^Yili Maternal and Infant Nutrition Institute, Inner Mongolia Yili Industrial Group, Co., Ltd, Beijing, China; ^4^Inner Mongolia Dairy Technology Research Institute Co., Ltd, Hohhot, China

**Keywords:** milk fat globule, lactation, human milk, maternal origin, phospholipids, fatty acids, gangliosides

## Abstract

There has been a growing interest in understanding how the relative levels of human milk fat globule (MFG) components change over the course of lactation, how they differ between populations, and implications of these changes for the health of the infant. In this article, we describe studies published over the last 30 years which have investigated components of the MFG in term milk, focusing on changes over the course of lactation and highlighting infant and maternal factors that may influence these changes. We then consider how the potential health benefits of some of the milk fat globule membrane (MFGM) components and derived ingredients relate to compositional and functional aspects and how these change throughout lactation. The results show that the concentrations of phospholipids, gangliosides, cholesterol, fatty acids and proteins vary throughout lactation, and such changes are likely to reflect the changing requirements of the growing infant. There is a lack of consistent trends for changes in phospholipids and gangliosides across lactation which may reflect different methodological approaches. Other factors such as maternal diet and geographical location have been shown to influence human MFGM composition. The majority of research on the health benefits of MFGM have been conducted using MFGM ingredients derived from bovine milk, and using animal models which have clearly demonstrated the role of the MFGM in supporting cognitive and immune health of infants at different stages of growth and development.

## Introduction

Given the importance of breastfeeding, with the WHO recommendation that infants be exclusively breast fed for the first 6 months of life (1), there is a growing interest in the health benefits of specific components of human milk (HM). There is also interest in understanding how the relative levels of these components change over the course of lactation, how they differ between populations, and implications of these changes for the health of the infant. In addition, with the low prevalence of breastfeeding, especially in high-income countries ( ≤ 40% at 6 months and ≤ 20% at 12 months) (2), knowledge on the compositional variation of HM helps to tailor new infant formulations to better meet the nutritional requirements of a growing infant.

**Figure 1:**
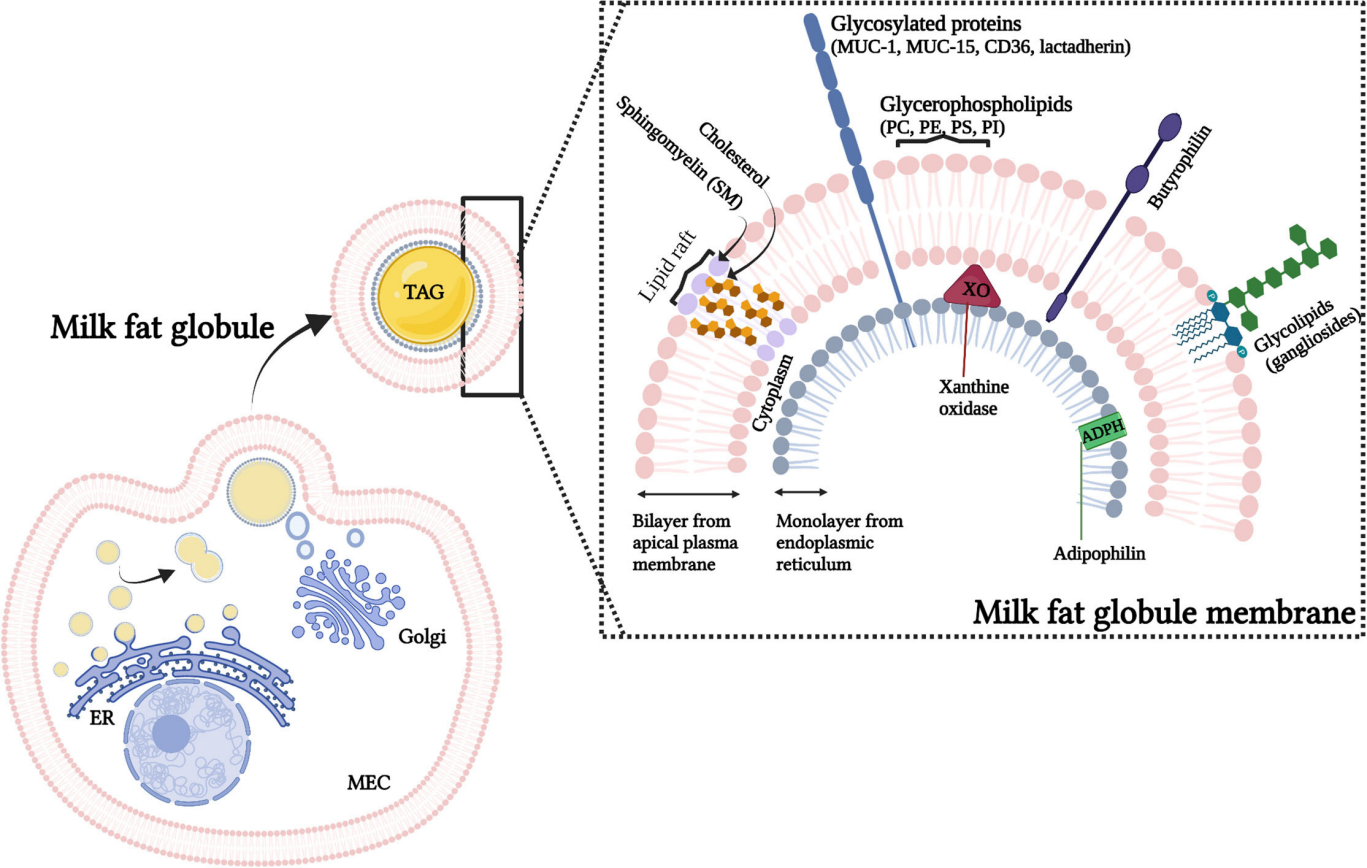
Representation of the origin and structure of the milk fat globules (MFG). MFG are secreted from the mammary epithelium cell (MEC), with components from the endoplasmic reticulum (ER), cytoplasm and cellular membrane. The secreted MFG contain a triacylglycerol (TAG) core and a tri-layered structure, the milk fat globule membrane (MFGM). The MFGM, in detail, shows the lateral segregation of sphingomyelin and glycolipids surrounded by the glycerophospholipids. Illustrative pictures of some of the major MFGM proteins (which includes mucins (MUC-1, MUC15), lactadherin, adipophilin (ADPH), xanthine oxidase (XO) and butyrophilin) are also presented. PC, phosphatidylcholine; PE, phosphatidylethanolamine; PS, phosphatidylserine; PI, phosphatidylinositol.

Fat is the component of HM that provides most of the energy and comprises a complex mixture of different lipid species (3). To enable this fat [including triglycerides, diglycerides, free fatty acids (FA), and cholesterol] to remain as a natural emulsion within milk, lipids produced within the secretory cells of the mammary gland are encapsulated by the milk fat globule membrane (MFGM) (Figure 1). As the milk fat globule (MFG) is synthesized in the rough endoplasmic reticulum, and transported through the cell cytoplasm, it is secreted through the apical membrane of the mammary epithelial cell. This results in the lipids being stabilized by a membrane with three distinct layers; an inner interfacial layer, the cytoplasm (enriched in protein), and finally a true bilayer membrane (4).

It has been proposed that the complex structure of the MFGM has arisen due to physiological constraints of the secretion process, and that it would not in itself be expected to contribute a significant health benefit to the offspring other than suppling the lipids necessary for growth and development (5). However, although some components of the MFGM are relatively minor within milk [for example, MFGM proteins contribute 1–4% of the total protein content in milk (6)], for others, such as phospholipids, gangliosides and cholesterol the MFGM represents the major source (7). Furthermore, although the fundamental physiological function of the MFGM is to allow for secretion of fat into milk, it is also clear that it communicates chemically important growth and immunological signals to the neonate (8). In addition, many different biological functions have been reported to be associated with MFGM proteins including protein synthesis/folding, signal transduction, transport, cell communication, as well as energy production metabolism, and immune function (6, 9).

In this article, we reviewed studies published from 1990 to 2020 that have investigated components of the MFGM in HM from mothers that delivered at term. The focuses were on those studies which have looked at changes over the course of lactation (at least two stages of lactation) and highlighted infant and maternal factors (including country of origin and diet) that may influence these changes. We then considered the functional and health effects of these compositional changes over the course of lactation. We also included a brief overview of the pre-clinical and clinical evidence of the health effects of MFGM components and we discussed whether MFGM is a necessary ingredient for infant formula products, to ensure that infants receive appropriate nutrition in the critical early years.

### Search Strategy, Study Selection and Exclusion Criteria

We performed a search and retrieved over 2,000 studies from Ovid Medline and Scopus databases from 1990 to 15th July 2021, as shown in Supplemental Material. All articles resulting from the search were assessed for eligibility based on the titles and abstracts. Of the remaining articles, full text was screened to check for eligibility. Only observational studies were included in this review. Studies were included if a specific human milk fat globule membrane component (phospholipids, proteins, gangliosides, and cholesterol) was measured at two or more time points during lactation period. For each milk component, we defined lactation period as colostrum (1 to 7 days post-partum), transitional milk (8 to 15 days post-partum) and mature milk (from 15 days post-partum). Studies reporting data from mothers that delivered at term were included. The reference lists of identified studies, reviews, and textbooks were reviewed to avoid missing relevant publications. Only studies in English were included. We created worksheets to systematically manage study selection, collating relevant study details such as date of publication, country of origin, lactation stage, measurement points, results, and confounding factors (method of collection, full expression, pre- or post-feed). We excluded (1) duplicate publications, (2) multiple publications of the same trial, (3) conference abstracts, (4) study protocols, (5) nonhuman studies, (6) studies and trials where any intervention was administrated to the mothers, (6) studies where samples were not from individual participants (pooled samples), and (7) studies reporting preterm milk data only.

## Milk Fat Globule

Milk fat is the most dynamic macronutrient in HM, and its yield affects the MFG size distribution and the composition and profile of MFGM components (10). The largest portion of milk fat consist of triglycerides (98%) in the form of MFG and other minor components such as diacylglycerides (<2%) and free FA (11). Total fat concentration increases during lactation, especially during the transition from colostrum to transitional milk (12, 13) with smaller changes from transitional to mature milk (12, 14). Although colostrum is known to be rich in immune factors and proteins, mature milk, in contrast, is energy dense to support infant growth (15). Fat concentration in colostrum, transitional and mature milk shows a increase trend from 1.1–5.9 to 3.0–5.6 g/100 and 2.0–6.1 g/100 mL, respectively, despite individual variability, sample type (pooled milk, full breast expression, foremilk, hindmilk) or sampling time (morning, night) (Supplementary Table 1, provides a summary of studies regarding total fat concentration in human colostrum, transitional, and mature milks).

Total fat changes during nursing; foremilk has a lower fat concentration compared to hindmilk (16, 17) and fat also follows a circadian rhythm (morning milk has lower concentration of fat compared to evening milk) (12). The effects of circadian rhythms in human fat have been recently reviewed (18). The authors reported that 15 out of 19 reviewed studies described circadian variation with the peak in the evening, for total fat concentration. Challenging this current dogma, a recent study reported that the concentrations of human foremilk fat, collected daily, from both breasts for 21 consecutive days did not differ according to time of day, day of week or breast used for collection (19). This suggests that circadian effects on milk fat may be observed only in hind milk (where the concentration of fat is higher) and that other factors, such as infant feeding pattern, time since last feed, breast fullness and lactation period may also play an important role on HM fat content. These factors may explain the large variation in fat concentration observed between mothers, and the consequent wide standard deviation reported in most studies (Supplementary Table 1).

The average MFG size can vary during lactation, due to the changes in the total amounts of milk fat produced during lactation (20). For example, it has been reported (21) that fat content as well as MFG average size increased from the third day (3.24 ± 1.68%, 3.77 ± 0.95 μm) until the 11 day of lactation (4.96 ± 2.13%, 5.09 ± 0.88 μm) and remained stable until the thirtieth day. In contradiction, others reported that whereas fat increased during lactation, colostrum had a larger MFG average size than transitional and mature milk (22, 23). This may be explained by coalescence of small globules with incomplete membrane coating provided by the immature mammary gland (24). It has also been demonstrated that increase in fat content in milk leads primarily to the increase in the number of MFG rather than the size (25). Changes in MFG numbers, therefore, are also likely to influence the concentration of MFGM components in milk. Overall, the increase in milk fat observed from colostrum to mature milk, independent of individual variability, may lead to changes in the MFG numbers and or size, affecting the concentration of membrane components in milk.

### Fatty Acids Composition

MFG (comprising a triacylglycerol core and the MFGM) are the main source of FAs in HM supplying not only energy but also essential and bioactive FAs for infant development. The HM FA profile is diverse with over 200 FA structures with different concentrations (26). Generally fatty acid compositional data from both the triacylglycerol core and the MFGM are reported as a total FAs profile.

**Figure 2:**
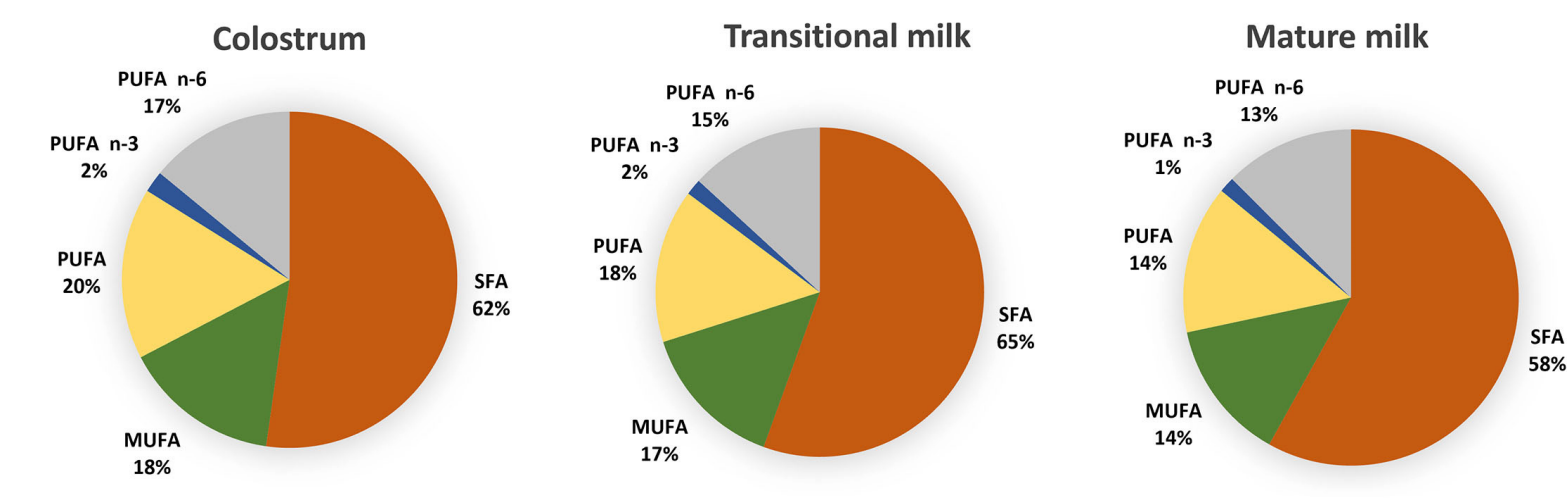
Distribution of fatty acids categories in colostrum (1–7 days), transitional milk (8–15 days) and mature milk (15–60 days) after pooled data analysis of worldwide milk samples reported by Floris et al. (28). (PUFAs) polyunsaturated fatty acids; (n-3 PUFAs) n-3 polyunsaturated fatty acids; (n-6 PUFAs) n-6 polyunsaturated fatty acids; (MUFA) monounsaturated fatty acids; (SFA) saturated fatty acids and (MCFA) medium chain fatty acids.

Saturated FA (SFA), monounsaturated FA (MUFA) and polyunsaturated FA (PUFA) represent 35–45%, 36–39 and ~18% of the total fat content of HM whereas short-chain FA (SCFA) and medium-chain FA (MCFAs) contribute relatively little (8%) (27, 28). Figure 2 reports fatty acid composition of pooled data analysis reported by Floris et al. (28). Three main sources of lipids are utilized in the synthesis of milk lipids: maternal dietary lipid, FAs from adipose tissue and *de novo* synthesized lipids. The importance of these sources is FA-specific and may influence their concentration at different stages of lactation. For example, palmitic acid (C16:0) and the essential FAs linoleic acid (LA, C18:2 n−6) and α-linolenic acid (ALA; C18:3 n−3) are sourced predominantly from maternal fat storage (70%) and only some from maternal dietary intake (30%) (29). MCFAs, however, are only synthesized *de novo* in the mammary gland, and the concentration have been suggested to be linked to mammary gland maturation and therefore present at higher concentrations in mature milk (30).

**Figure 3:**
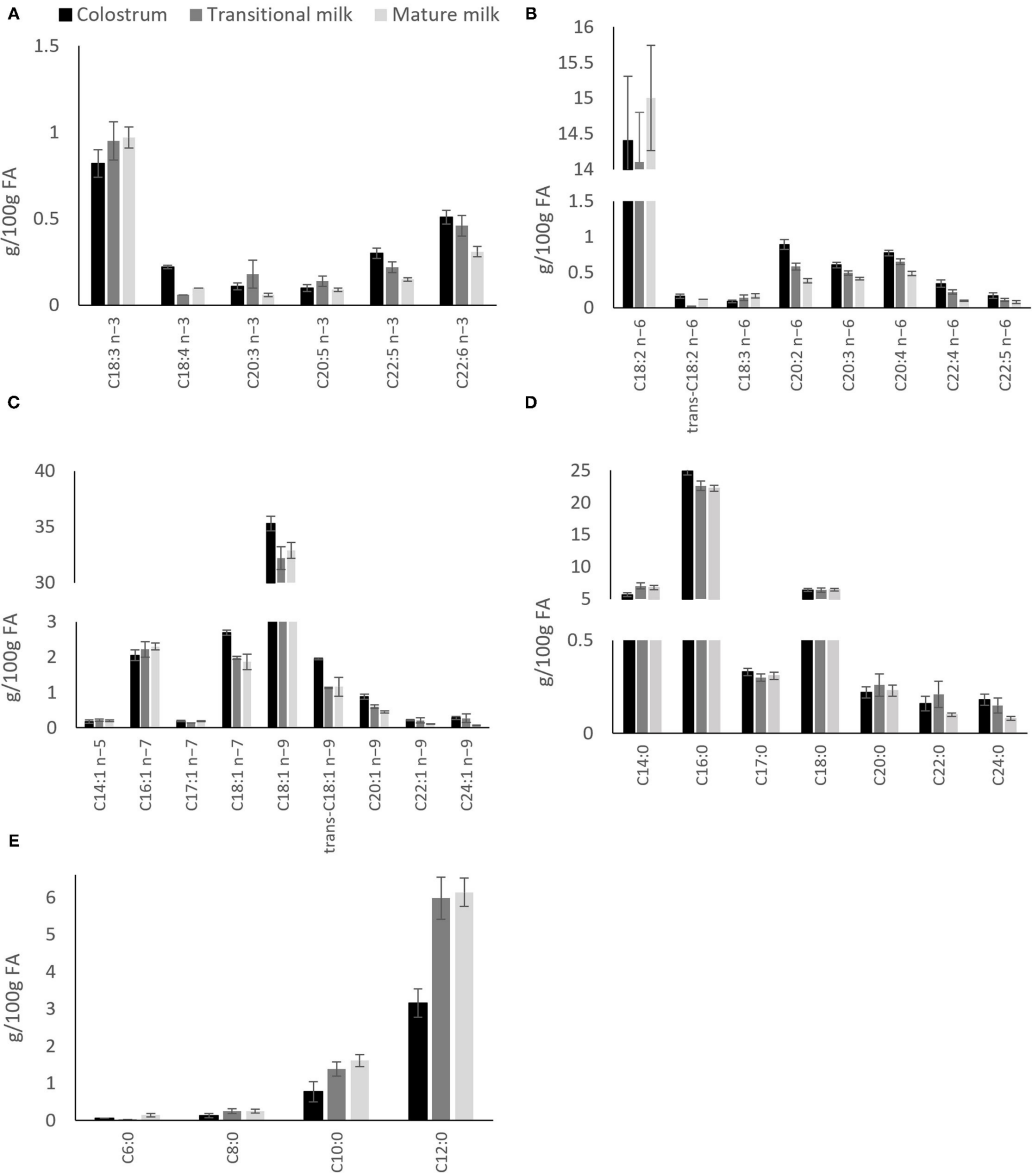
Distribution of individual fatty acids in colostrum, transitional milk and mature milk after pooled data analysis of worldwide milk samples reported by Floris et al. (28). **(A)** n-3 polyunsaturated fatty acids; **(B)** n-6 polyunsaturated fatty acids; **(C)** Monounsaturated fatty acids; **(D)** Saturated fatty acids and; **(E)** Medium chain (C6-C12) saturated fatty acids.

A recent systematic review including data from 55 studies worldwide, and a total of 4,374 term milk samples reported analysis for the variation of 36 main FA across lactation (28). The most abundant SFA, palmitic (C16:0), stearic (C18:0) and myristic (C14:0) and MUFA oleic acid (C18:1 n-9) were shown to remain stable whereas gondoic (C20:1 n−9), erucic (C22:1 n−9) and nervonic (C24:1 n−9) acid were shown to decrease over the course of lactation (Figure 3) (28). The opposite pattern was observed for the MCFAs, specially the most abundant, lauric acid (C12:0), which was shown to almost double from colostrum to transitional milk. Among the long-chain PUFAs, n−6 PUFAs, and more specifically LA (C18:2 n−6, 16%), were the most abundant FA compared to n−3 PUFAs (3%). LA, eicosapentaenoic (EPA; C20:5 n−3) and ALA concentrations were shown to be relatively stable during lactation (Figure 3). However, a steady decrease of arachidonic acid (ARA; C20:4 n−6), docosahexaenoic acid (DHA, C22:6 n−3) and docosapentaenoic acid (DPA; C22:5 n−3) over the course of lactation has been consistently reported in other literature (28, 31–33) (Figure 3).

### Factors Affecting Fatty Acid Profile

**Figure 4:**
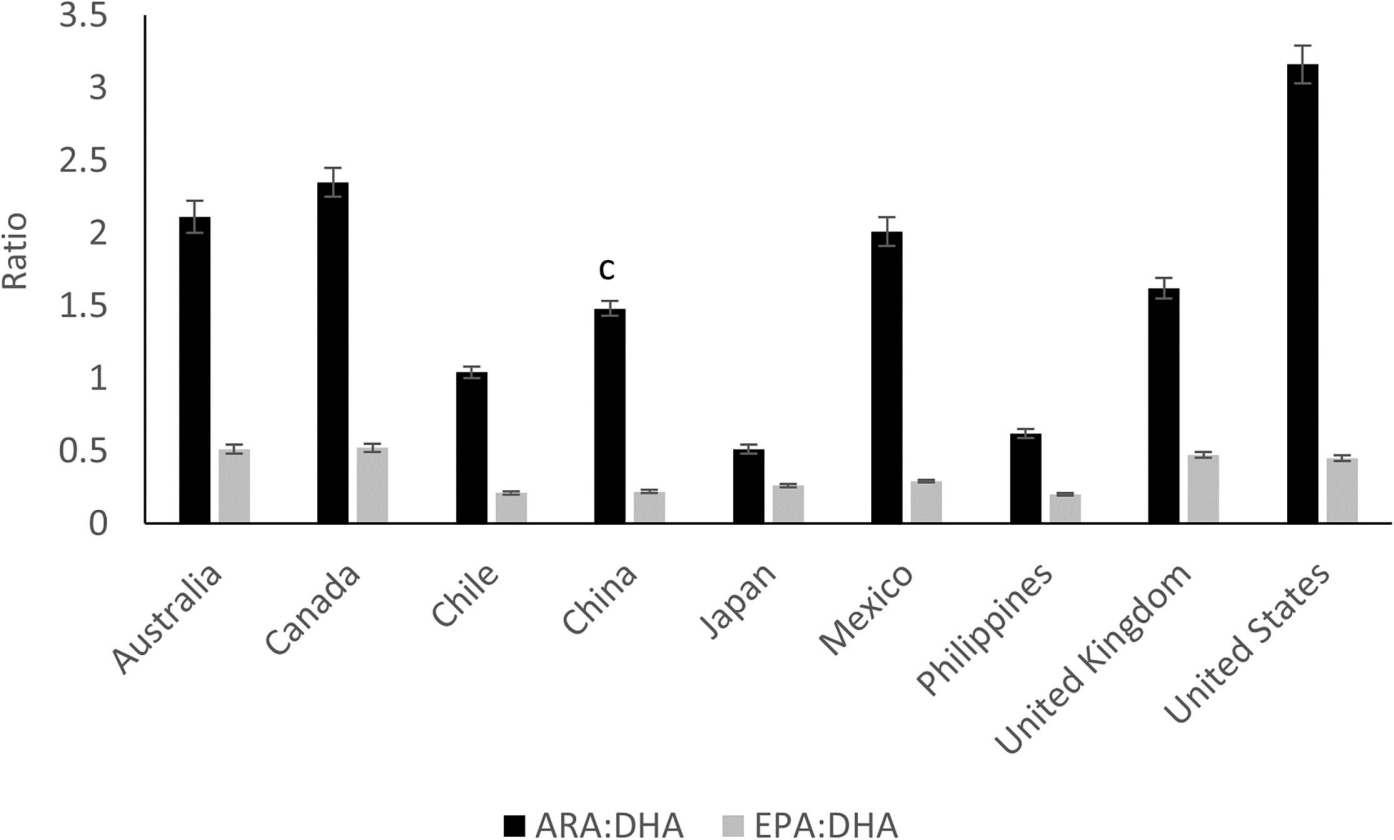
The ratio of arachidonic acid to docosahexaenoic acid (ARA:DHA) and the ratio of eicosapentaenoic acid to docosahexaenoic acid (EPA:DHA) in mature human milk from nine different countries. Data extracted from Yuhas et al. (44). Means with different superscripts are statically different (*P* < 0.05), *n* = 44–54, per country.

Numerous studies have indicated the influence of maternal origin, ethnicity (34), diet and cultural habits on the composition of milk FAs (35–41). Brenna et al. (42) in a meta-analysis of 65 studies worldwide found that DHA concentration varies greatly among countries with a mean (±SD) concentration of 0.32 ± 0.22 and a range of 0.06–1.4%. DHA levels were higher in countries with high consumption of fish, such as the Canadian Artic (1.4%) and the Philippines, Japan (43, 44), Chile (44, 45) and Taiwan (46) with levels above 0.4%. In contrast, countries such as Pakistan (0.06%), Canada and the United States, had very low DHA levels (below 0.2%). Geographical locations with the highest DHA levels, e.g., Philippines and Japan also had the highest EPA (C20:5n-3) levels ranging from 0.15–0.26% (44) (Figure 4). Compared to DHA, ARA levels were shown to be less variable among countries, with the mean level of all samples being 0.41% (44) (Figure 4). This may due to the poor conversion of dietary LA to ARA in milk (47). The mean ARA:DHA ratio found for most countries (Australia, Canada, UK, Mexico, China, Spain) was 1.6:1 whereas the ratio was lower for Japan (0.5:1) and higher for China (2:1 to 3:1) (35, 36, 48) and USA (3.2:1) (44) (Figure 4). Although optimal ARA:DHA ratios are not fully elucidated, the ratio of both LCPUFAs was suggested to impact immune response, cognitive and behavioral outcomes, competition for tissue incorporation and risk for atopic disease (49–51).

Human Milk LA levels were found to be relatively elevated in countries, such as Mexico and Chile (~16%) that, typically consume a high-maize diet, compared to Australia, Canada, Iran and the UK (~10%) (44, 45, 52, 53). High levels of HM LA (22%) and ARA (0.5%) were also reported in China compared to Sweden (10 and 0.3%, respectively) (36), which may be linked to the high consumption of SFA by the Chinese population (54). The Philippines presented an odd FA profile, with low LA levels (7%) and high levels of lauric (13%) and myristic acids (12%) (44) compared to other Asian countries (~5 and 3–6%, respectively), which may indicate consumption of diets restricted in both total fat and essential FA (55). The FA profile from Canada and China (~35%) had the highest oleic acid content compared to UK, Australia, Japan, Chile and Mexico (26–32%) (44), most likely due to a relatively elevated intake of canola or rapeseed oils in these countries (56, 57). The ALA levels were constant, at ~1% of FA for Australia, Canada, Chile, Japan, UK, USA, Iran and Mexico, but lower for the Philippines (0.43%) and higher for China (2%) (44, 53). In general, higher percentages of PUFA have been reported in Chinese HM studies (22.4–30.0%) (41, 58–60) compared to developed Asian countries (17.25–21·50%) (44, 61) and those in the Western countries (11.5–21%) (23, 62).

It is important to note that studies generally report data from a specific country region, and interpretation of data must be related to that specific geographical area and not to the entire country. Previous studies have demonstrated that several countries, such as France (63) and China (64) have regional differences. It is also worth noting that factors other than diet and geographical location may affect the profile of FA in milk. LA, for example, was shown to be consistently more highly expressed in HM secreted for male infants (37% increase) compared to female (65). The effect of gestational age on FA profile has been reviewed and linked to changes in DHA concentration (increase in premature milk) in some studies (66) but not in others (28).

## Composition of the Milk fat Globule Membrane

The composition of the MFGM is more intricate than the MFG core, with a ratio of ~1:1 of proteins and lipids (67). Other minor components, such as RNA, are also present (68). The major MFGM proteins (i.e., that are well-described, and are present in relatively high concentrations) are mucin 1 (MUC 1), xanthine oxidoreductase (XDH/XO or XOR), butyrophilin (BTN), lactadherin (PAS 6/7, MFG-E8), CD 36, adipophilin, and fatty acid-binding protein (FABP) (69). Proteomics studies have demonstrated that there are at least 200 (70, 71) and perhaps more than 400 (71, 72) proteins within the MFGM of HM, and their relevance for human health is an area of active research and commercial interest. In this review we focus on a number of studies in which the putative role of some of the major proteins are reported, and we also include more detail on some proteomics studies of the human MFGM, however it is beyond the scope of this review to consider the levels and function of these minor proteins in detail.

The key lipid species of the MFGM are phospholipids, with phosphatidylethanolamine (PE, 6–36%), phosphatidylcholine (PC, 14–38%), and sphingomyelin (SM, also a sphingolipid, 27–43%) being the major species, and phosphatidylserine (PS) and phosphatidylinositol (PI) are relatively minor components (**Table 2**) (58, 73). Other important lipid components of the MFGM are cholesterol, gangliosides and FA, which appear to play an integral role in cognitive development (58).

### Phospholipids

Milk phospholipids have important functional properties influencing general lipid absorption (74, 75), brain development (76, 77) gut mucosal development (78), and immune maturation (79).

Table 1 provides a summary of studies of total phospholipid content of HM across lactation. Total phospholipids appear to vary through lactation, with some studies reporting a decrease over time (7, 58, 81, 83–86), whereas in one study the total milk phospholipids concentration was reported to not vary across lactation (82). Other studies have reported an increase in total phospholipids from colostrum to transitional milk followed by a decrease over the mature milk period (13, 23, 84). These results might be explained by the relationship between the phospholipids contents and the diameter of the MFG, where phospholipids are generally negatively correlated to the diameter of MFG (transitional milk < mature milk < colostrum). For a constant total fat content in milk, more phospholipids are required to cover the larger surface area of smaller MFG (see details in “Milk Fat Globule” section).

**Table 1:** Total phospholipid concentration in human colostrum, transitional, and mature milks.

**Mothers**	**Units**	**Colostrum**	**Transition milk**	**Mature milk**					**References**
**Country**		**(Day 1–7)**	**(Day 8–15)**	**1 month**	**2 months**	**3 months**	**4 months**	**Up to 8 months**	
Singapore	mg/100 mL			23.0 ± 4.9	20.8 ± 8.5		24.2 ± 8.2		(65)
China (Beijing)	mg/100 mL	33.0 ± 11.2	24.4 ± 8.1					22.3 ± 9.9	(58)
China (Suzhou)	mg/100 mL	38.9 ± 18.8	34.9 ± 16.6					26.02 ± 11.3	(58)
China (Guangzhou)	mg/100 mL	33.2 ± 8.1	25.6 ± 11.1					25.3 ± 12.5	(58)
China (Shanghai, Huangpu)	mg/100 mL		40.7			22.9			(80)
China (Shanghai)^*^	mg/100 g	35.1 ± 10.8	35.1 ± 8.6		28.1 ± 7.8	–			(81)
China (Wuxi)	μmol/100 mL	25.8 ± 3.8	24.8 ± 3.5	23.7 ± 2.4	23.6 ± 3.6	22.6 ± 1.1			(82)
Ireland (Cork)	mg/100 mL	67.7 ± 14.5	48.7 ± 18.1					36.9 ± 16.4	(7)
France (Marseilles)	mg/100 mL	72 ± 51	55 ± 26	45 ± 26					(83)
Denmark	mg polar lipids/100 g total lipids	4.4 ± 0.4	5.9 ± 0.3	5.1 ± 0.4					(23)
Spain (Granada)	nmol/mL	202 ± 39	209 ± 38	147 ± 23					(84)
Spain (Madrid)	mg/100 mL	37.2 ± 1.0	43.7 ± 2.3	39.2 ± 2.8	35.9 ± 0.9		26.5 ± 0.7		(13)
Spain (Valencia)	mg/100 mL	31.5 ± 3.1	53.5 ± 2.5	42.2 ± 1.3		32.1 ± 1.8		33.4 ± 2.0	(13)
Spain (Murcia)	mg/100 mL			39.2 ± 2.5		34.9 ± 2.0		28.1 ± 1.4	(13)
United Arab Emirates (Sharjah, Dubai, and Ajman)	mg/L	NR	269.0 ± 89.2					219.6 ± 85.0	(85)
Malay	mg/L	352.4 ± 166.3	273.0 ± 58.4		147.1 ± 41.2			187.5 ± 110.0	(86)

* *Only measured PC, PE, and SM. NR, not reported*.

*The concentration is shown as mean ± standard deviation. Units differ according to each publication*.

The effects of geographical location on total HM phospholipid composition were evaluated by Claumarchirant et al. (13), reporting a higher concentration of total phospholipids in transitional milk and at 6 months after delivery in a geographical coastal zone (Valencia, 33–53 mg 100 mL^−1^) compared to the central zone (Madrid, 26–43 mg 100 mL^−1^) of Spain. Similarly, the geographical differences in total phospholipid in colostrum among Chinese cities (Beijing, Suzhou and Guangzhou) has also been reported recently by Giuffrida et al. (58). This study showed higher concentrations of total phospholipid in mothers from Suzhou (38 mg 100 mL^−1^) compared to the other cities (33 mg 100 mL^−1^) (Table 2). The authors hypothesized that these differences could be due to the increased consumption of marine foods or rapeseed oil in this region.

**Table 2:** Concentration of phospholipid species in human colostrum, transitional, and mature milks.

**Colostrum (Day 1–7)**												
References		(81)	(82)	(84)	(23)	(7)	(58)	(58)	(58)	(86)	(13)	
Country		China (Shanghai)	China (Wuxi)	Spain (Granada)	Denmark	Ireland (Cork)	China (Beijing)	China (Suzhou)	China (Guangzhou)	Malay	Spain (Madrid)	Spain (Valencia and Murcia)
Units		mg/100 g	μmol/100 mL	Total (202 ± 39 nmol/mL)	mg polar lipids/total lipids	mg/100 mL	mg/100 mL	mg/100 mL	mg/100 mL	mg/L	mg/100 mL	
				Weight%								
PE		4.61 ± 2.11	7.39 ± 0.58	5.86 ± 0.63	0.41 ± 0.03	49.40 ± 13.68	7.6 ± 3.1	12.6 ± 7.4	9.9 ± 2.6	89.9 ± 25.8	12.36 ± 0.61	10.46 ± 0.75
PC		20.32 ± 6.61	7.55 ± 1.52	38.40 ± 3.09	1.26 ± 0.19	11.44 ± 2.64	10.9 ± 4.8	12.6 ± 7.7	12.5 ± 4.6	76.7 ± 55.4	4.87 ± 0.11	4.51 ± 0.28
PS			1.29 ± 0.09	7.91 ± 1.12	0.56 ± 0.03		1.8 ± 2.3	1.7 ± 0.5	1.3 ± 0.4	125.8 ± 63.0	3.49 ± 0.01	3.43 ± 0.20
PI			1.05 ± 0.06	6.03 ± 0.61	0.36 ± 0.02		1.6 ± 0.5	2.3 ± 1.0	1.8 ± 0.5	11.2 ± 3.3	3.13 ± 0.01	3.12 ± 0.15
SM		10.14 ± 3.39	8.54 ± 1.83	40.49 ± 3.57	1.82 ± 0.26	6.90 ± 1.26	10.9 ± 4.9	9.7 ± 3.1	7.7 ± 1.6	39.7 ± 25.7	13.36 ± 0.29	9.95 ± 1.75
**Transition milk (Day 8–15)**												
References	(58)	(58)	(58)	(81)	(23)	(82)	(84)	(7)	(85)	(86)	(13)	
Country	China (Beijing)	China (Suzhou)	China (Guangzhou)	China (Shanghai)	Denmark	China (Wuxi)	Spain (Granada)	Ireland (Cork)	United Arab Emirates	Malay	Spain (Madrid)	Spain (Valencia and Murcia)
Units	mg/100 mL	mg/100 mL	mg/100 mL	mg/100 g	mg polar lipids/total lipids	μmol/100 mL	Total (209 ± 38 nmol/mL)	mg/100 mL	mg/L	mg/L	mg/100 mL	
							Weight%					
PE	7.3 ± 2.4	10.8 ± 5.8	5.6 ± 3.7	4.79 ± 2.01	0.77 ± 0.12	7.07 ± 0.60	8.55 ± 1.16	37.86 ± 14.00	66.3 ± 27.16	100.0 ± 24.5	13.90 ± 0.98	15.90 ± 0.85
PC	8.3 ± 3.7	11.9 ± 6.1	11.3 ± 5.6	19.94 ± 5.35	1.50 ± 0.13	7.21 ± 1.20	37.69 ± 4.88	6.56 ± 3.26	66.4 ± 32.87	48.6 ± 11.6	5.97 ± 0.15	8.09 ± 0.14
PS	1.0 ± 0.4	1.3 ± 0.5	0.8 ± 0.4			1.21 ± 0.24	8.17 ± 1.04		28.5 ± 13.29	90.9 ± 18.0	4.66 ± 0.10	6.74 ± 0.08
PI	1.5 ± 0.4	2.4 ± 1.1	1.2 ± 0.7		0.40 ± 0.03	0.93 ± 0.30	5.21 ± 0.54		11.2 ± 5.5	9.6 ± 3.0	4.30 ± 0.07a	6.32 ± 0.05
SM	6.2 ± 3.8	8.5 ± 4.7	6.8 ± 2.7	10.37 ± 2.69	2.37 ± 0.40	8.37 ± 1.54	39.20 ± 3.63	4.23 ± 1.88	91.2 ± 26.38	20.9 ± 5.7	14.86 ± 1.11	16.49 ± 1.46
**Mature milk**												
**1 month**							**(16–60 days)**			**2 months**		
References	(23)		(82)	(82)	(84)	(65)	(13)			(81)	(82)	(86)
Country	Denmark		China (Wuxi)	China (Wuxi)	Spain (Granada)	Singapore	Spain (Madrid)	Spain (Valencia)	Spain (Murcia)	China (Shanghai)	China (Wuxi)	Malay
Units	mg polar lipids/total lipids		μmol/100 mL	μmol/100 mL	μmol/100 mL	mg/100 mL	mg/100 mL			mg/100 g	μmol/100 mL	mg/L
PE	0.76 ± 0.10		7.21 ± 1.20	6.92 ± 0.98	1.87 ± 0.17	6.76 ± 1.86	11.98 ± 1.09	12.68 ± 0.66	11.58 ± 0.98	3.63 ± 1.53	6.89 ± 1.22	39.3 ± 15.8
PC	1.07 ± 0.11		1.21 ± 0.24	6.74 ± 0.72	4.59 ± 0.70	5.97 ± 1.34	5.42 ± 0.31	6.55 ± 0.13	5.67 ± 0.24	15.41 ± 5.09	6.06 ± 0.60	21.0 ± 10.8
PS	0.84 ± 0.06		0.93 ± 0.30	1.03 ± 0.15	1.52 ± 0.19	0.75 ± 0.31	4.45 ± 0.24	5.55 ± 0.07	4.90 ± 0.18		1.15 ± 0.22	14.8 ± 7.7
PI	0.41 ± 0.02		8.37 ± 1.54	0.64 ± 0.08	0.86 ± 0.07	1.07 ± 0.35	4.15 ± 0.24	5.20 ± 0.04	4.60 ± 0.17		1.00 ± 0.21	6.3 ± 3.6
SM	1.97 ± 0.33		41.03 ± 3.41	8.34 ± 0.94	6.03 ± 0.50	8.47 ± 1.72	13.20 ± 1.27	12.19 ± 0.54	12.44 ± 1.11	9.07 ± 2.52	6.89 ± 1.22	57.4 ± 11.7
**Mature milk**												
**3 months**					**61–135 days**					**4 months**	**6 months**	
References		(65)		(82)		(13)			(7)		(87)	
Country		Singapore		China (Wuxi)		Spain (Madrid)	Spain (Valencia)	Spain (Murcia)	Ireland (Cork)		Malay	
Units		mg/100 mL		μmol/100 mL		mg/100 mL			mg/100 mL		mg/L	
PE		6.36 ± 3.11		7.13 ± 0.32		10.71 ± 0.24	9.51 ± 0.76	11.36 ± 0.73	29.15 ± 13.04		66.1 ± 0.	
PC		4.84 ± 2.06		5.89 ± 0.53		5.06 ± 0.14	4.92 ± 0.13	4.86 ± 0.16	4.50 ± 1.97		23.7 ± 0.	
PS		0.75 ± 0.33		0.85 ± 0.07		4.56 ± 0.14	4.39 ± 0.06	4.30 ± 0.13			15.5 ± 9.4	
PI		1.13 ± 0.55		0.75 ± 0.14		4.23 ± 0.11	4.13 ± 0.05	4.06 ± 0.13			5.9 ± 0.0	
SM		7.71 ± 3.01		8.15 ± 0.55		11.34 ± 0.34	9.20 ± 0.90	10.29 ± 0.83	3.29 ± 1.73		70.4 ± 36.8	
**Mature milk**												
**Up to 8 months**												
References		(65)		(85)			(58)			(13)		
Country	Singapore	United Arab Emirates (Sharjah, Dubai, and Ajman)	China (Beijing)	China (Suzhou)		China (Guangzhou)		Spain (Madrid)		Spain (Valencia)		Spain (Murcia)
Units	mg/100 mL	mg/L		mg/100 mL		mg/100 mL		mg/100 mL		mg/100 mL		
PE	8.08 ± 3.10	80.0 ± 35.35	5.3 ± 2.6	7.3 ± 3.2	7.1 ± 3.9		8.29 ± 0.40		10.24 ± 0.60		8.37 ± 0.56	
PC	4.94 ± 1.88	30.2 ± 22.07	7.6 ± 4.5	8.5 ± 5.3	8.6 ± 5.1		3.79 ± 0.19		5.02 ± 0.16		4.13 ± 0.12	
PS	0.91 ± 0.33-	16.1 ± 6.99	0.9 ± 1.2	1.2 ± 1.4	1.0 ± 0.6		3.39 ± 0.17		4.49 ± 0.07		3.73 ± 0.11	
PI	1.67 ± 0.66	6.5 ± 3.61	1.2 ± 0.5	1.7 ± 0.8	1.5 ± 0.8		3.12 ± 0.1		4.23 ± 0.08		3.50 ± 0.09	
SM	8.26 ± 2.64	82.9 ± 29.21	7.3 ± 3.9	7.4 ± 4.2	7.1 ± 4.0		7.96 ± 0.11		9.44 ± 1.09		8.39 ± 0.52	

*PE, phosphatidylethanolamine; PI, phosphatidylinositol; PS, phosphatidylserine; PC, phosphatidylcholine; SM, sphingomyelin*.

*The concentration is shown as mean ± standard deviation. Units differ according to each publication*.

Table 2 provides a summary of studies on the variation of HM phospholipid species across lactation. SM was found to be the most abundant phospholipid (27.4–43.4% of total phospholipid) from analysis using Phosphorus-31 nuclear magnetic resonance (^31^P NMR), Thin-layer chromatography (TCL) (43.3 ± 2.6%) (82, 88) and by High-performance liquid chromatography with evaporative light-scattering detection (HPLC-ELSD) (23, 65, 84), with the exception of Giuffrida et al. (58) who reported PC as the most abundant phospholipid using ELSD. HM phospholipids analyses using liquid chromatography–mass spectrometry generally conclude that the major phospholipid is PE (7, 89). The difference in phospholipid composition may be explained by the response differences of the detectors, or by other factors, such as diet, geographical location, sampling time, and gestation age at birth (preterm vs. term), metabolic stage, and diurnal rhythm. Supplementary Table 2, summarizes the methodology used to collect and analyses the phospholipid composition in the studies reviewed.

**Figure 5:**
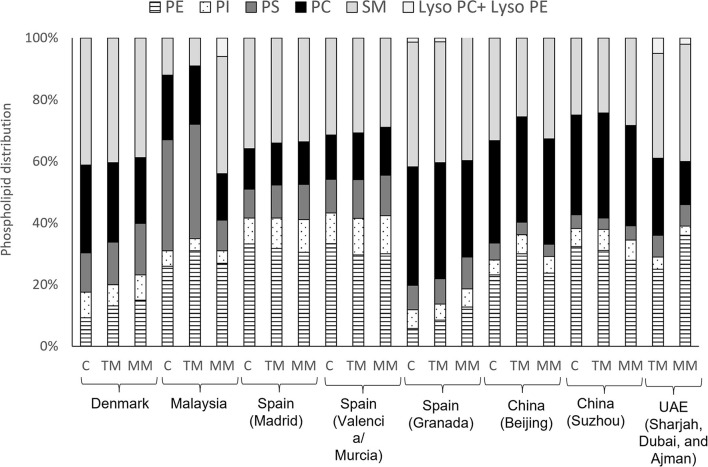
Distribution of phospholipid classes in colostrum (C), transitional milk (TM) and mature milk (MM) in different geographical cohorts. Data from (23), Denmark; (86), Malaysia; (13), Spain (Madrid, Valencia and Murcia); (84), Spain (Granada); (58), China (Beijing and Suzhou); (85), UAE (Sharjah, Dubai, and Ajman). PE, phosphatidylethanolamine; PI, phosphatidylinositol; PS, phosphatidylserine; PC, phosphatidylcholine; SM, sphingomyelin.

Some differences in the distribution of HM phospholipid classes have also been reported in different geographical locations (Figure 5). A study conducted in a Chinese population showed higher proportions of PC (35%) and lower concentrations of PE (26%) in mature milk (58), compared to mothers from the Unites Arab Emirates [14 and 36%, respectively (85)], Spain [Madrid, Valencia and Murcia (15 and 31%, respectively) and Malaya (14 and 36%, respectively (86)]. The studies conducted in Spain showed that the distribution of mature milk phospholipids from Granada (84) had higher proportions of PC (38%) and lower proportions of PE (6%) compared to other parts of Spain (Madrid, Valencia and Murcia, 13 and 32% respectively) (13) (Figure 5). These discrepancies may be due to the type of sample collection, as one study collected hindmilk (84) and another did not report the type of sample collected (13) (Supplementary Table 2).

Variation in phospholipid classes during lactation is shown in Table 2. PC, PI and PS concentration was reported to be elevated in colostrum, and decreased to lower levels in transitional and mature milk (82) whereas, no significant differences were found in the concentration of PE (82, 85) or SM (82, 84, 85) during lactation. Other studies (7, 58, 81), however, found that all individual PL decreased from colostrum to mature milk. An increase in PE during lactation was observed by Sala-Vila et al. (84). These authors reported an increased ratio of PUFA to saturated fatty acid from colostrum and mature milk, suggesting that this variation is related to the evolution of the fatty acid content of total phospholipids. This increase may be due to the preferential pattern of distribution of FAs into the different classes of phospholipids. Although SM mainly esterifies mainly saturated and monounsaturated fatty acids (MUFAs), PUFA are mainly esterified in PE. PE exhibits an increase in LA (C18:2 n-6) as lactation progresses from the secretion of colostrum to transitional milk and then to mature milk (90).

#### Phospholipids Fatty Acids Composition

**Figure 6:**
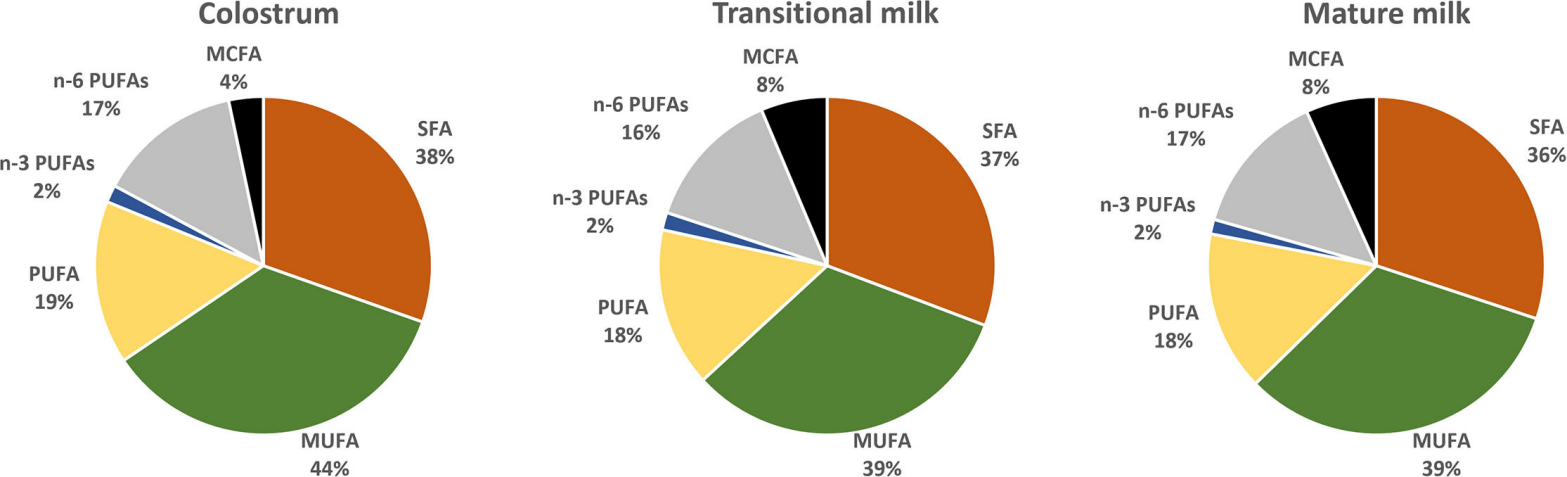
Distribution of phospholipids fatty acids categories in colostrum (1–7 days), transitional milk (8–15 days) and mature milk (60 days) from Chinese mothers reported by Wei et al. (82). PUFAs, polyunsaturated fatty acids; n-3 PUFAs, n-3 polyunsaturated fatty acids; n-6 PUFAs, n-6 polyunsaturated fatty acids; MUFA, monounsaturated fatty acids; SFA, saturated fatty acids.

There are only a few studies that investigated the FAs composition associated with phospholipids in HM (23, 84, 88, 89, 91). Compared to a total milk FAs profile the MFGM had increased concentrations of SFAs at all lactation stages (23). The SFA (mainly C16:0 and C18:0) represent around 60–70% of FAs, followed by oleic acid (C18:1 n-9), LA (C18:2 n-6) and the LC-PUFA ARA (C20:4,n-6), adding up to 80% of total FAs (7, 82, 84, 91). Mature milk was reported to contain higher amounts of saturated MCFA and lower contents of C16:0 compared to colostrum and transitional milk, whereas the contents of total MUFAs and PUFAs were not different (23) (Figure 6). PUFAs (n-3) were found to increase from colostrum to mature milk, whereas no difference was found for n-6 PUFAs, especially for C18:2 n-6 (23). Another study found no difference among total SFAs, MUFAs and PUFAs at different lactation stages but an increase in C18:2 n-6 in mature milk (84). This is important as the degree of FAs unsaturation, together with cholesterol and SM, influence membrane fluidity. SFAs allow the phospholipids to pack more closely in the membrane decreasing fluidity, whereas unsaturated FAs increase fluidity (92), and affecting digestion and perhaps functionality (93). The high content of SFA and LCFAs (C22:0, C24:0 and C18:0) were shown to contribute to the structural role of SM, maintaining rigidity of the MFGM (94).

Although ARA and DHA are mainly found in a triglyceride structure within the core of the MFG, they are also found in MFGM phospholipids, principally in PE (7, 89). One study reported that around 10% of ARA is found in the phospholipid fraction for both transitional and mature milk, whereas ~10 and 22% of the DHA was found in the phospholipid fraction in transitional and mature milk, respectively (83). This same study also reported that the DHA:ARA ratio was significantly higher in the phospholipid fraction compared to the triacylglycerol core, suggesting that HM with the higher phospholipid concentrations may be more efficient for brain and intestinal LC-PUFA accretion since phospholipids provides a best delivery system (95, 96).

Recent studies, using a lipidomic approach, described the distribution of HM glycerophospholipids molecular species across lactation (7, 97, 98). The major glycerophospholipids molecular species for PE, PC, PI and PS found in mature milk of a Chinese cohort was C36:2 (35–64%) followed by C36:1 [16–28%, with the exception of PC (4%)], with both phospholipids increasing during lactation (98). This may indicate that similar FAs moieties (34–36 carbons) across the range of glycerophospholipids may have a functional role in the MFGM. Similarly, high concentrations of C36:2 for PE were also found in the milk of Singaporean mothers (97), whereas C36:4 was the major molecular specie reported from Irish mothers (7). The major types of PC varied according to the study with C36:2 and C32:0 representing 31–41 and 10–13% of the molecular species, respectively, in some studies (97, 98), while in another, it was about 12 and 46%, respectively (7).

SM has a very distinct molecular profile in mature milk, with d40:1 (20%), d42:1 (16%), d36:1 (14%), d34:1 (13%), d42:2 and d38:1 (10% each), adding up to 85% of total SM molecules (98). Although these major SM molecules were identified in other studies (91, 97), their distribution were different. A very different profile of SM molecules, d38:0 (47%), d38:1 (13%), d40:1 (16%), d32:1 (7%), and d40:0 (13%), was reported in an Irish cohort (7). As previously discussed, FAs composition can be affected by maternal dietary factors and maternal geographical origin observed across different cohorts. To our knowledge, no study has addressed the effects of maternal geographical origin and diet on phospholipid FAs, only on the total fatty acids profile.

### Gangliosides

Table 3 provides a summary of studies reporting total concentrations of HM gangliosides and GD3 (disialoganglioside) and GM3 (monosialodihexosylganglioside) molecular structures across lactation. Higher concentrations of total gangliosides were found in colostrum, followed by transitional milk with the lowest in mature milk, in most studies (99, 102, 105, 106). A few studies reported that the concentrations of gangliosides were relatively consistent during lactation (100, 101) or increased from colostrum to mature milk (58, 103) and continued to increase in mature milk (87, 106). Such discrepancies in the results could be due to differences in sampling, analytical methods (Supplementary Table 2) and other factors such as maternal diet and infant gender, etc. A significant positive correlation between gangliosides and total milk lipid has been described (87, 100, 104, 106). Gangliosides are found in the MFGM surrounding the fat droplet; therefore, if the gangliosides were a constant proportion of the MFGM and the MFG were a similar size, more gangliosides would be expected with an increase in fat content.

**Table 3:** Concentration of ganglioside molecular structures in human colostrum, transitional, and mature milks.

	**Colostrum**								**0–11 days**		
References	(99)	(79)^*^	(100)	(100)	(101)^*^	(87)^#^	(102)	(58)^#^	(103)^#^	
Country	Spain	Spain	Panama	Spain	Japan	Malaysia	China	China	China	
Units	mg LBSA/kg	μg LBSA/g	μg LBSA/g	μg LBSA/g	μg LBSA/mL	mg/L	mg/L	mg/L	μg/mL	
Total	2.3 ± 0.5	2.9	2.2 ± 0.4	3.6 ± 0.6	9.2 ± 2.0	26.8	15.9 ± 5.9	8.0 ± 5.3	8.1	
GM_3_	8.8 ± 2.2%	7.40%	6.2 ± 2.3%	6.2 ± 2.3%	3.7 ± 2.0%	6.5 ± 7.0	2.3 ± 0.5	3.8 ± 2.5	4.3 ± 0.9	
GD_3_	**63.2** **±** **4.0%**	**37.40%**	**51.3** **±** **7.0%**	**43.7** **±** **2.5%**	**46.7** **±** **9.9%**	**20.3** **±** **13.0**	**13.7** **±** **5.7**	**4.1** **±** **4.5**	**3.8** **±** **0.4**	
	**Transitional milk**									**16 days-8 months**
References	(99)	(79)^*^	(100)	(100)	(101)^*^	(87)^#^	(102)	(58)^#^	(103)^#^	(99)
Country	Spain	Spain	Panama	Spain	Japan	United Arab Emirates	Malaysia	China	China	China
Units	mg LBSA/kg	μg LBSA/g	μg/g	μg/g	μg LBSA/mL	mg/L	mg/L	mg/L	mg/L	mg/L
Total	1.38 ± 0.4	1.54	3.8 ± 0.8	3.7 ± 0.6	9.2 ± 0.2	21.2 ± 11.5	18.9	12.7 ± 4.5	8.5 ± 4.5	11.0 ± 5.0
GM_3_	11.6 ± 2.2%	1.6	12.0 ± 2.0%	10.2 ± 1.6%	26.7 ± 5.9%	9.5 ± 8.4	8.3 ± 4.8	2.1 ± 0.3	5.5 ± 3.2	10.1 ± 4.6
GD_3_	52.4 ± 4.2%	21	34.4 ± 3.5%	38.9 ± 3.0%	31.2 ± 7.6%	11.7 ± 9.5	10.6 ± 4.3	10.6 ± 4.4	3.0 ± 3.4	1.0 ± 1.7
	**Mature milk**									
	**1 month**									
References	(101)^*^	(104)^*^	(100)	(100)	(42)	(103)^#^	(58)^#^	(65)^#^	(102)	(86)
Country	Japan	Spain	Panama	Spain	Spain	China	China	Singapore	China	China (Guangzhou)
Units	μg LBSA/ mL	μg LBSA/g	LBSA μg/g	LBSA μg/g	mg LBSA/kg	μg/mL	mg/L	mg/L	mg/L	mg/L
Total	9.0 ± 1.6	0.82	4.3 ± 2.1	2.1 ± 0.5	0.8 ± 0.2	9.1			9.5 ± 3.1	13.1 ± 6.7
GM_3_	32.1 ± 7.6%	47.4%	27.2 ± 6.5%	22.0 ± 2.7%	50.2 ± 1.6%	7.4 ± 0.2	10.1 ± 4.6	2.3 ± 0.8	2.2 ± 1.1	8.5 ± 4.7%
GD_3_	19.9 ± 2.1%	8.2%	23.2 ± 6.9%	21.5 ± 3.7%	21.3 ± 1.3%	1.7 ± 0.2	1.0 ± 1.7	2.3 ± 1.2	7.2 ± 3.2	4.6 ± 3.1%
	**Mature milk**									
	**2 months**					**3 months**				
References	(101)^*^	(104)^*^	(100)	(100)	(42)	(103)^#^	(58)^#^	(65)^#^	(102)	
Country	Spain	China (Guangzhou)	China	China	Singapore	Malaysia	Spain	China	China (Guangzhou)	
Units	μg LBSA/g	mg/L	mg/L	μg/mL	mg/L	mg/L	μg LBSA/g	mg/L	mg/L	
Total	0.85	18.2 ± 7.8	7.4 ± 2.2	10		14.8 ± 8.0	1.39	7.5 ± 2.3	20.9 ± 10.5	
GM_3_	42.3%	11.3 ± 6.2%	2.2 ± 0.8	9.1 ± 0.3	2.9 ± 1.4	8.3 ± 5.5	56.3%	2.1 ± 0.6	17.4 ± 9.0%	
GD_3_	9.3%	7.0 ± 7.7%	5.2 ± 2.1	0.9 ± 0.1	1.9 ± 2.0	6.5 ± 5.1	13.5%	5.3 ± 2.3	3.5 ± 2.5%	
	**Mature milk**									
	**4 months**					**6 months**				
**References**	**(** **65** **)** ^#^	**(** **102** **)**	**(** **103** **)** ^#^	**(** **87** **)**	**(** **87** **)**	**(** **87** **)** ^#^	**(** **102** **)**	**(** **85** **)** ^#^	
Country	Singapore	China	China	China (Guangzhou)	China (Guangzhou)	Malaysia	China	United Arab Emirates	
Units	mg/L	mg/L	μg/mL	mg/g	mg/g	mg/L	mg/L	mg/L	
Total		7.0 ± 2.5	10.7	19.8 ± 9.4	22.9 ± 9.9	25.3 ± 15.7	6.5 ± 2.0	20.2 ± 9.8	
GM_3_	3.9 ± 1.8	2.1 ± 0.6	9.8 ± 0.3	18.3 ± 9.4%	21.4 ± 9.5%	21.4 ± 13.0	2.1 ± 0.8	18.6 ± 9.7	
GD_3_	1.7 ± 1.9	4.9 ± 2.4	0.9 ± 0.1	1.5 ± 1.1%	1.5 ± 1.1%	4.3 ± 5.5	4.4 ± 1.9	1.6 ± 2.2	

* *Colostrum (5 days post-partum), Transitional milk (10 days postpartum). LBSA, lipid bound sialic acid*.

# *Total as sum of GM3 and GD3*.

*The concentration is shown as mean ± standard deviation. Units differ according to each publication*.

*LBSA, lipid bound sialic acid. ^#^Total as sum of GM3 and GD3*.

Due to the large range of ganglioside structures, quantification of gangliosides can be difficult. Until 2009, conventional methods to detect and quantify gangliosides were mainly based on high-performance thin-layer chromatography (HPTLC) and results were converted from lipid-bound sialic acid (LBSA). Data were often inaccurate due to limitations of these method (107). An improved HPLC-MS method was developed by Fong et al. (108), reporting the content and number and a large number of ganglioside structures in different food matrixes. In 2013–2014, this method was used to report that the total content of gangliosides in HM of Singaporean and Chinese mothers 30–120 days after delivery was 4.6–5.6 and 9.1–10.7 mg/L, respectively (65, 103). In 2015, Ma et al. reported the content of HM in mothers from South China within 8 months after delivery as 13.1–22.9 mg/L (106) and Tan et al. (102) reported the content of gangliosides in HM of Chinese mothers was 6.5–15.9 mg/L within 6 months after delivery (102). The lowest content (0.8 mg/L) was observed for Spanish samples from studies using the HPTLC methodology (99). It is interesting to note that high overall ganglioside concentrations in milk were reported in Asian mothers (7- 25.3 mg/L) (Table 3) (58, 101, 102, 106) where nutritional aspects (such as fat being mainly sourced from fish) may largely contribute to these findings.

Among the seven different gangliosides that have been identified in HM (99, 101, 104), GD3 and GM3, referred to as “simple” gangliosides, are the prevalent individual components of the ganglioside fraction. GD3 ganglioside is the predominant form present in human colostrum and transitional milk (30–80%) but concentrations decrease up to 4–6 months post-partum (8–25%). Conversely, GM3 is predominance in mature milk (58, 100, 106, 109). These gangliosides are likely to survive the infant's digestion, reaching the intestinal tract and having an inhibitory effect on the adhesion of pathogenic bacteria (110, 111). It has been suggested that in early milk GD3 may have a role in organ development, such as of the gut and brain (112). The increase in GM3 in mature milk has been linked to the development of the immune and central nervous systems by supporting signal transduction, cell adhesion, and growth factor receptors (79). Therefore, the variation in the ganglioside composition of HM over the course of lactation might be linked to alterations that occur in the immunological prophylactic system, and in the development of the central nervous system and the autonomic nervous system of the intestine and other organs.

Large variations in the concentration of individual gangliosides can be observed in studies using similar methodologies (Supplementary Table 2). Giuffrida et al. (58) reported average GM3 values, in colostrum and transitional milk, of 4.1 and 3.0 mg/L, respectively, whereas Ma et al. (87) reported 20 and 10 mg/L, respectively. Within mature milk, at 1–2 months and 3–8 months GD3 content was reported to be as low as 0.87 and 0.25–0.50 mg/L, respectively (58) and high as 4.6–7.0 and 1.5–2.7 mg/L (87, 106), respectively in Asian mothers. Interestingly, infant gender may influence gangliosides concentration with one study reporting an increase in GM3 in milk for male infants at 120 days of lactation compared to milk for female infants (65). These authors indicated that the increase in the total amount of lipid in milk for male infants at 120 days (119%) could partly explain the observed increase in GM3 and other amphipathic molecules such as phospholipids (PC, PI, PE, and SM).

Only one study compared the concentrations of HM gangliosides in different countries across lactation stages (100). Although no statistically significant differences were observed across locations and lactation periods, the gangliosides content tended to be higher in Spanish mothers colostrum compared to Panamanian mothers colostrum. The opposite observation was found in mature milk, where fat and gangliosides content were enriched in Panamanian mothers (100). These authors indicate that although different dietary habits were observed among these countries, the use of foremilk for their investigations may have masked the effects of maternal origin on gangliosides content.

### Cholesterol

The MFGM is the source of cholesterol in HM, which is essential for the synthesis of lipoproteins, bile acids, hormones and calciferols, therefore, essential to infant growth (113). Moreover, cholesterol is a crucial part of the cell membranes and myelin, and is especially required during the neuroplasticity period (from conception to up to 4 years old) (114, 115). Despite the importance of cholesterol, only a few studies have examined the concentration of this bioactive compound in HM during lactation. Most studies, not reviewed here, were published before 1990 and showed large variability, probably due to the limitations of analytical methods available at the time.

In general HM cholesterol changes dynamically throughout lactation, with the highest level in colostrum, decreasing significantly during the first month after delivery (116–118) (Table 4). One particular study showed that HM cholesterol decreased by 60% from colostrum to the first month postpartum (118), and another by half at 6 months postpartum (123). After the first month postpartum, the decline in cholesterol concentration was shown to be much less pronounced (117, 118, 123) and this may be associated with the MFG size and number. Changes in the MFG diameter from colostrum to mature milk (from ~ 3μm in colostrum to around 5 μm in mature milk) as well as a decrease in the number of globules leads to reduced MFGM surface area and consequently, cholesterol.

**Table 4:** Concentration of cholesterol in human colostrum, transitional, and mature milks.

**Country**	**Sample collection**	**Method analysis**	**Colostrum**	**Transitional**	**Mature milk**						**Reference**
					**1 month**	**2 months**	**3 months**	**4 months**	**5 months**	**6 months**	
Spain	Pooled milk	E-S			11.3 ± 0.4						(119)
Iraqi	Partial expression (5–10 mL) of two breasts combined morning	E-S	28.3 ± 4.2								(120)
USA	NR	GC						14.2 ± 3.3			(121)
Netherlands	24 h sample	GC-FID		16.6			12.8 ± 1.0				(122)
Spain	Pooled milk	GC	20.7 ± 0.6	14.8 ± 0.8	12.8 ± 0.5		10.9 ± 0.5		10.1 ± 0.4		(123)
		E-S	23.2 ± 1.1	17.1 ± 0.8	13.6 ± 0.5		12.8 ± 0.2		11.7 ± 0.1	
Poland	NR	ATR-FTIR	3.4–11.9	4.4–13.0							(124)
Portugal	NR	HPLC-DAD	29.2 ± 0.01		17.4 ± 0.5	12.0 ± 0.1		9.5 ± 0.1			(117)
African	Manual expression, mid-way through nursing	GC	36.0 ± 16.2	19.7 ± 0.7	19.0 ± 0.8						(116)
China	Full single breast expression, mechanical expression, morning	HPLC	20	17.1	12.6						(118)

*NR, not reported; E-S, enzymatic–spectrophotometric; GC, gas chromatography; ATR-FTIR, attenuated total reflectance–Fourier transform infrared spectroscopy; HPLC-DAD, high-performance liquid chromatography with diode array detector*.

*The concentration is shown as mean ± standard deviation (mg/100 mL)*.

Studies of HM in Iraq, Spain, Portugal, and China reported similar ranges of cholesterol in colostrum (20–29 mg/100 mL), whereas studies in Poland and Africa reported the lowest (3.4–11.9 mg/100 mL) and the highest (36.0 ± 16.2 mg/100 mL) concentrations, respectively. Similar results were observed for mature milk, with cholesterol concentrations ranging from 11 to 13 mg/100 mL for most countries but higher in the study conducted in Africa (19 mg/mL).

### Proteins

Proteins represent 25–60% of the total MFGM mass and 1–4% of the total protein content of HM. The use of proteomic techniques has enabled the assessment of MFGM-derived proteins to understand their diversity and physiological roles (70–72, 125–129). Beyond a nutritional source, the main human MFGM proteins were shown to have a role on cell communication and signal transduction, immune function, metabolism, and energy production (6).

The proteomics studies generally do not provide absolute quantitative data, however by comparing relative levels they can provide information on how MFGM proteins change over the course of lactation. In one relatively early study, Cavaletto et al. (130) used proteomics to assess the MFGM butyrophilin (BTN) protein family (which comprises seven proteins); they observed only slight differences in BTN spot distribution when comparing colostrum with mature milk. More recently, relative quantification of MFGM proteins during lactation was performed by label free spectral counting and differentiation expression analysis (6). This demonstrated a change in relative levels of many minor MFGM proteins from early to late lactation; for example, alpha-1-antitrypsin, alpha-amylase, apolipoproteins D and E, alpha-enolase, insulin-like growth factor-binding protein 2 and long chain fatty acid-coA ligase 4 were expressed at higher levels during early lactation (particularly in colostrum), whereas CD9 antigen, fatty acid binding protein, folate receptor alpha, and glutathione peroxidase 3 were expressed at higher levels during late lactation (6–12 months). Interestingly, other proteins such as xanthine dehydrogenase/oxidase, complement C3, BTN A1 and Annexin 2 had a sharp increase in concentration later in lactation (3–6 months). Other studies showed that, as observed for total proteins (131), the colostral MFGM proteome contains a higher number of proteins related to the establishing immune system than mature milk (71, 129). This may indicate that proteins may be expressed to aid a particular developmental stage of the infant (73).

#### Individual MFGM Proteins

Within the timeframe specified for this review, there are few reports in which levels of MFGM proteins have been quantified in HM. One study of 45 mothers in León, Nicaragua measured lactadherin in HM at 3 months of age, reporting a median concentration of 5.4 μg/mL (interquartile range, 4.0–7.3) (132). It should be noted that this level was measured following a rotavirus vaccination, so it is not clear whether this represents a normal level in HM. In addition, there were no data on changes in lactadherin through lactation in this study.

In an observational study, 200 infants in Mexico were recruited at birth, and their stool monitored for rotavirus infection; at the same time, samples of mothers' milk were collected, and assayed for a range of MFGM-associated proteins (133). Similar to observations regarding levels of phospholipids, these analyses showed wide inter-individual variation, with levels of lactadherin in HM ranging from 5.6 to 180 μg/mL. This also demonstrated an association between levels of lactadherin and protection of infants from rotavirus infection, consistent with this protein playing a role in immunity and response to infection.

#### Glycoproteins and Phosphoproteins

Protein glycosylation, the attachment of a carbohydrate (glycan) to a protein, is one of the most common post-translational modifications of proteins. Glycosylation has been reported to be involved in several biological and cellular functions, including protein folding, immune response, and pathogen binding (134, 135). Many MFGM proteins, for example, the major proteins mucins, lactadherin, and butyrophilin (136). LC-MS/MS analysis of pooled samples from a total of 60 mothers (30 colostrum, 30 mature milk) identified 220 MFGM N-glycoproteins differentially expressed in mature milk compared with colostrum, demonstrating a significant shift in N-glycoprotein composition of HM across lactation (72). Among those proteins differently glycosylated, the proteins involved in immune system maturation and microbial colonization, such as lactoperoxidase, major histocompatibility complex (MHC) and cell adhesion molecules (CAMs) showed increased N-glycosylation levels in colostrum compared to mature milk (72). This may play a significant role in the formation of the immune system of infants. Also, it has been proposed that not only the overall concentration of a protein is crucial for its overall activity but understanding glycosylation pattern during lactation could also reflect the individual needs of infants during their growth (137).

Protein phosphorylation is another common posttranslational modification, regulating various cellular processes such as protein location, interaction, and overall function (138). Therefore, understanding the variation in phosphopeptides is very important to recognize the changes of many biological processes in health and disease (139). Recently, a quantitative phosphoproteomics analysis of human MFGM demonstrated that colostrum and mature milk have different phosphorylation profiles (129). Among 203 phosphoproteins identified, 48 proteins were differentially expressed between the different stages of lactation. Of those, phosphoproteins related to the cellular process and immunity (27 and 24 phosphorylation sites, respectively) were identified only in human colostrum milk.

## Factors Influencing MFGM Components

Stage of lactation is clearly one factor that impacts on MFGM composition. There are also several maternal factors influencing MFGM content of HM, and these have recently been reviewed (17, 73). Some of them, such as stage of lactation, circadian rhythms, infant birth weight, gender (140), development at delivery (pre-term vs. term) (141), maternal diet and weight, and method of breast milk expression (17) can directly affect the total lipid content in milk, and MFG size and numbers. Because lipids and proteins are the major components of the MFGM, these have been most widely assessed in this context.

### Lipids

Although FAs in the core of the globule and those in the MFGM have not been separately analyzed in many studies of milk lipids (73), some insight has been gained into key maternal factors that influence the lipid composition of the MFGM. In addition to lactation stage, the following factors also influence MFGM-derived lipid levels in HM.

#### Method of Sample Collection

There are two main methods of HM collection, by hand or mechanical expression by electric pump. An electric pump cycles the negative pressure with a rhythmic action simulating suckling, which provides a standardized method to collect milk samples (142). Milk hand expression generally requires breast massage, which can increase release of milk fat (143). Variation in nutrient content across expression methods needs to be considered when interpreting data.

#### Time of Collection and Subsampling

Lipid content varies over the course of the day (with higher concentrations found in the evening milk) and ideally milk samples representative of 24h production should be obtained. The difficulty to obtain these samples generally leads to the collection of full expression from one breast, a few mL of hindmilk, foremilk or a combination of both (142). The total amount of fat changes over time during nursing; foremilk has a lower fat concentration compared to hindmilk (16, 17) and fat also follows a circadian rhythm (morning milk has lower concentration of fat compared to evening milk) [reviewed by Italianer et al. (18)]. It has also been suggested that nursing frequency and breast (left or right) may affect milk macronutrient composition. The impact of breast (left or right) on macronutrient composition has been linked to the level of fullness of the breast before sampling, in turn linked with the last feed (144). This highlights the importance of standardization of milk collection methods or at least a thoughtful reporting of the conditions of milk collection in studies that report milk composition data.

#### Genetic Factors

Few studies have been published on maternal genetics regulating levels of MFGM phospholipid classes. One such study describes a polymorphism in the diacylglycerol acyltransferase 1 (DGAT1) gene which was associated with altered phospholipid composition and phospholipid/TAG ratios (145). However, this study was on bovine milk, hence more research is needed to understand the extent of the influence of genetic variation in HM.

#### Diet

Of particular relevance to this review, milk ganglioside, FAs and phospholipid concentrations have been reported to differ according to geographical locations, suggesting that diet may influence the amounts in HM (58). Indeed, several studies have reported associations between maternal diet and milk lipid composition [reviewed by Bravi et al. (146)]. Crossover (147–149) and observational studies (146, 150) indicate that maternal lipid intake plays an important role on the HM total fat content and FA profile. Lipids from maternal diet is one of the three known sources of milk lipids, the other being *de novo* synthesis and FAs from maternal adipose tissue. Other food components, such as choline dietary supplementation has been shown to be positively correlated with HM PC, especially in choline-deficient diets (151). The impact of maternal diet, however, may vary for particular MFGM components. The content of the long-chain fatty acid DHA within particular phospholipids in HM, for example, appears to be independent of the maternal intake of these compounds (152).

#### Gender of the Infant

The potential for the infant's gender to influence maternal milk composition has recently been reviewed (153). Evidence to support this idea is largely from animal studies which suggest gender is a predictive determinant of milk composition. In human studies, the lipid content in mature milk produced for males was higher than milk produced for female infants (65, 154). Authors hypothesized that higher suckling response (longer and more frequent) from male infants may feedback as a message for additional energy content that results in increased energy output from the mother. It is important to note that most studies did not assess individual milk production or infant intake, restricting the ability to account for volume and overall fat production.

#### Gestation Length (Term vs. Pre-term)

There is evidence that pre-term delivery may result in a different phospholipid profile in HM (7), with some evidence that sphingomyelin and PE may decrease in full-term colostrum, whereas other phospholipids such as PC, PI, and PS showed no correlation with delivery term (155). However, as this review is focused on normal, term infants we have not considered this aspect further.

#### Maternal Factors

Maternal factors such as body weight have been shown to correlate with milk lipid concentration in early (20, 39, 156) and late lactation (after 6 months post-partum) (157, 158). In a recent systematic review of 63 datapoints, a meta-regression analysis demonstrated a positive association between maternal BMI and human milk fat (115). It has been hypothesized that while in early lactation fat stores accumulated during pregnancy are mobilized for milk production. Later in lactation, where fat accumulated during pregnancy is depleted, the effects of maternal weight (and blood triglyceride concentration) may become more apparent (157).

Maternal age was shown to affect milk volume, with an average fall of up to 40% in the yield of breastmilk from the age of 20 to 30 years and above, as reported in several studies (159–161). Dewey et al. (160), hypothesized that milk yield is dependent on the amount of functional breast tissue, which may decrease with age due to atrophy. Interestingly, maternal age was shown to affect fat concentration in colostrum, which was increased in mothers over 35 years old compared to younger mothers, but not in transitional or mature milk. Remarkably, maternal weight was similar between the groups leading to the hypothesis that changes in maternal metabolism with age may be linked to the observed results (162, 163). Other maternal factors such as tobacco smoking (164) was associated with a lower content of milk lipids, while no link between exercise (165) and maternal genetic factors on changes in milk fat concentration have been reported (166).

### Proteins

In addition to stage of lactation, environmental influence is one of other key factors affecting proteins within the MFGM. As stated above, MFGM proteins showed various functions, such as immune defense, leading to health benefits that will be described further in following section. Therefore, fluctuations in MFGM immune-related proteins were observed as part of immune response during environmental challenges, e.g., bacterial infection. MFGM proteins that are up-regulated in response to such a challenge include those involved in host defense, inflammation, and oxidative stress. It should be noted that these observations are from other mammal species such as cows and sheep, however similar phenomena likely occur in human MFGM. Whether those changes have implications for the breast-feeding infant is not yet clear (73).

## Health Benefits of the MFGM

The health benefits of MFGM have recently been reviewed (167, 168). There is strong evidence to demonstrate several health benefits derived from the MFGM, including cognitive and immune function, gut health and maturation, metabolism (including cholesterol and insulin metabolism) and even skin health. Some of these benefits, such as mobility, have been investigated exclusively in the context of aging humans, with a focus on the potential development of functional foods to enhance the health of aging population. There are several benefits of relevance for the growing infant, including immune and cognitive development and function, and gut maturation and health.

Most of evidence suggesting health benefits of the MFGM come from preclinical and clinical studies testing the effects of purified MFGM components or ingredients from bovine milk. Commercially available MFGM ingredients are extracted from bovine buttermilk, beta serum or whey producing products with different total composition (lactose, protein, ash, total lipids and phospholipids) and the distribution of phospholipid species (Table 5). Although beta serum provides the highest concentration of phospholipids (≥14%), whey offers a high concentration of phospholipids (7.5%) to be used as supplement and provides an excellent source of protein (73%), especially for infant formula supplementation. The profile of proteins in MFGM-enriched dairy products are not usually described in product label information, however, it is likely that products sourced from cream, beta serum and buttermilk contain MFGM protein as well as other components like gangliosides, cholesterol, lactoferrin, sialic acid and IgG.

**Table 5:** The composition of commercially available MFGM-enriched dairy ingredients compared to mature human milk.

	**Bovine MFGM enriched ingredient**			**Mature human milk**
**Concentration in product (g/100 g)**	**Buttermilk^#^**	**Beta serum^#^**	**Whey^#^**	
Lactose	± 50	≤ 10	≤ 3	
Protein (N × 6.38)	≥30	>52	73	
Ash	≤ 9	≤ 6	≤ 3	
Total lipids	5–13	3–27	12–26	4 (range 2.0–6.1)
Total phospholipids (PL) (g/100 g of fat)^*^	1.6–22	≥14	5–16	0.5–1
**Phospholipids (% of total PL)**				
Phosphatidyl ethanolamine (PE)	35–43	22–29	19–41	12–36
Phosphatidyl choline (PC)	19–32	27–47	19–25	13–34
Phosphatidyl serine (PS)	8–18	1.2–23	8–12	4–16
Phosphatidyl inositol (PI)	4–9	1–8	3.6–7	3–12
Sphingomyelin (SM)	11–19	14–27	16–24	28–41
Gangliosides (g/100 g of fat)^*^	NR	NR	NR	0.02–0.05
Cholesterol (g/100 g of fat)^*^	NR	NR	NR	0.2–0.48

* *Based on the variation of milk fat content average of (4 g/100 mL), phospholipid (23–43 mg/100 mL), gangliosides (0.7–2.0 mg/100 mL) and cholesterol (11–19 mg/100 mL) in mature milk described in this review*.

# *Data compiled by Fontecha et al. (168)*.

Most proteins in human and bovine MFGM are from the same classes such as BTN, ADPH, FABP, MUC1, XDH and lactadherin (MFGE8). BTN was shown to be the most abundant protein, in both bovine (24.8%) and human (16.3%) MFGM (71). The main difference between human and bovine MFGM proteins is that human MFGM has a higher level of proteins involved in immune response and in lipid catabolism than bovine MFGM (169). Human MFGM, for example, is enriched in immunoglobulins whereas bovine MFGM is enriched in antimicrobial proteins (131). However, the MFGM proteome and protein functionalities between the two species are mostly similar suggesting that MFGM proteins could have a positive impact in infant health such as anti-adhesive and antimicrobial functions (169).

The lipid profile and the distribution of phospholipid species of bovine MFGM enriched ingredient and HM are described in Table 5. The large variation found in lipid composition in mature HM demonstrate the potential of this ingredients to be tailored to supplement IF with polar lipids, closing the gap between breast milk and infant formula composition. Although it is important to note that the phospholipid fatty acid profile differ between human and bovine milk (170), the tolerance and beneficial effects of dietary bovine milk polar lipids has been described in many clinical studies, recently reviewed by Brink and Lonnerdal (167).

In the following sections, we briefly summarize the key evidence of the health benefits of MFGM (including mechanistic studies in animals, and studies in human populations) and discuss how changes in specific components across lactation may reflect the particular role of these components in the health of the growing infant.

### Cognitive Function

Cognitive function is the most widely studied potential benefit of the MFGM, because of the rapid cognitive development that occurs during early human life (in particular gestation and lactation) and the consequent influence on cognitive function throughout life. So far, five clinical trials have been published reporting the effects of dietary bovine MFGM or MFGM components (through supplemented infant formula) on neurological development (171–175). Although it is generally acknowledged that more research is needed in infants, the evidence supports the hypothesis that there are cognitive (173, 176), neurodevelopmental (171, 172, 174, 177–179) and vision functional (175) benefits from MFGM for infants. This evidence is also supported by pre-clinical studies where individual components of MFGM, or diets enriched with bovine MFGM derived product were fed to various animal models.

Gangliosides and sialic acid may be the active components within the MFGM mediating cognitive effects, possibly by ensuring sufficient amounts of these nutrients are available for the developing brain (76, 180, 181), although there is also evidence that these compounds when supplied exogenously may influence growth signaling in the brain leading to improvements in learning and (spatial) memory outcomes (76, 177, 182, 183). It is well established that there are key periods during human brain development when there is rapid accumulation of particular lipids, for example gangliosides in the forebrain at 32 weeks of gestation and plasmalogens in both cerebrum and cerebellum from 32 weeks of gestation to 6 months of age and beyond (184). Both plasmalogens and gangliosides are components of the MFGM, associated with essential processes [myelination and synaptic development, respectively (184)] for appropriate growth and development of the brain.

Phospholipids, especially choline (which is found attached to the phosphate group of PC) sourced during pregnancy were associated with improved infant cognitive scores (176) and neurodevelopmental outcomes (178, 179). Choline is a nutrient that affects DNA methylation, long term potentiation and neural cell populations in the hippocampus as demonstrated in fetal rats (185–187). Its role in postnatal brain development is assumed to be equally important as postnatally, as its concentration in human milk was shown to be 15 times higher than in maternal blood (188).

PUFAs, in particular ARA and DHA, have been extensively studied for their impact on brain development. Pre- and postnatal development of infant brain and retina, for example, require a rapid accumulation of long-chain PUFAs (189). Healthy brain tissue consists of about 60% structural fat; of this, about 25% is DHA and 15% ARA (190, 191). Postnatally ARA and DHA are supplied mostly by human milk and, to some extent, by the infant's adipose tissue (192) influencing the PUFA profile of infant blood and tissue (193). ARA and DHA can be synthesized by chain elongation and desaturation of essential FAs, such as LA (C18: 2n-6) and ALA (C18: 3n-3), however, in infants, due to low enzymatic activity, this synthesis is very low (194) and influenced by genetic heritability (195). Thus, dietary intake of ARA and DHA are essential for infant's brain development.

It is important to understand the relevance of changes in components such as these across lactation for cognitive development, as these changes may reflect particular times at which these components may influence infant development.

### Immune Function

Although not as well researched as the impact on cognitive function, there have been reports on the effects of MFGM components on immune function. Human studies have largely been in infants and relate to prevention from infection by micro-organisms (both bacteria and viruses) (196–198), with a large body of *in vitro* and animal studies investigating mechanisms of action and potential active molecules (199–202). In one clinical trial, bovine MFGM supplementation in infant formula was found to reduce febrile episodes in infants and young children and the number of days with fever among 2.5 to 6 year old children (203). Dietary supplementation of bovine milk complex milk lipids (197) or whey-derived MFGM (protein rich) (196), lowered the duration (197) and/or incidence of diarrhea (196) in infants. Reduction of the incidence of respiratory illness was also reported as a result of bovine MFGM supplementation in infants (174).

Much of this research relates to the ability of a range of MFGM components such as gangliosides (204, 205), sialic acid (206), proteins [butyrophilin, lactadherin, and fatty acid binding protein (207)], and glycoproteins to act as decoys for pathogens and therefore prevent infection. The reducing effects of MFGM on the expression of *E. coli* virulence gene (202) and the ability to bind to human epithelial cells (208) were suggested as a possible mechanism to prevent infection. However, there is also good evidence of other mechanisms, including neutralizing viral and bacterial toxins [gangliosides (209)], direct toxicity to the invading organisms, or prevention of growth and invasion [XDH (210, 211), MUC 1 and 4 (212)]. The lipid component of the MFGM and its digestion products were shown to have bactericidal activity against rotavirus (213) and food borne pathogens (214).

There is also evidence of MFGM components having immune-modulatory effects such as influencing cytokine production and macrophages (215, 216), although these effects are not as well characterized as those relating to protection from pathogens.

### Gut Maturation and Gut Health

Studies have shown that MFGM components may have an impact on the development of the gut epithelium and immune system, however, these findings largely come from pre-clinical studies in animal models, in particular pigs, rats, and mice during the pre-weaning period, which is critical for gut maturation.

Intestinal maturation being influenced by dietary bovine MFGM was shown in piglets and rats, with specific effects being improvement of intestinal morphology, increased enzymatic activity, and reduction of the proportions of pathogenic bacteria (78, 217). *In vivo* studies showed that MFGM components purified or extracted from bovine milk protect the gut from injury [for example by carcinogens (199) and bacterial lipopolysaccharide (LPS) (218)]. These results also suggest there is a role for MFGM on gut maturation. Phospholipids in particular were shown to protect the gut from injury in a dextran sodium sulfate challenge model (213) and reduced the depletion of goblet cells by decreasing the overactivated Notch signaling pathways (219). In a lipopolysaccharide challenge model, bovine MFGM supplementation to suckling mouse pups decreased epithelium injury, inflammatory cytokines and increased the expression of gap junction proteins (220–222). The MFGM protein lactadherin was shown to support wound healing by binding to intestinal cell *in vitro* (223). These studies indicate that MFGM components are able to support the development of the infant intestine by directly strengthening, protecting and up regulating the intestinal barrier.

As summarized by Rueda (79), dietary gangliosides may play various roles relating to gut development and health, including modifying the microbiota, influencing the development of the gut immune system, and modulation of oral tolerance during early life. Overall, they appear to promote gut immunity development in the neonate, and consequently play a role in the prevention of infections during early infancy. Other MFGM components also clearly have an impact on gut maturation, affecting factors such as villus height, cell maturation, and gut enzyme activity (for example, lactase) (224).

### Gut Microbiota

In addition to being important in gut maturation, barrier function and resilience and modulation of inflammation, the MFGM appears to have beneficial effects through promotion of a beneficial gut microbiota (78). To date, only one clinical study showed direct effect of bovine whey-derived MFGM on the oral microbiome of formula fed infants, with the species *Moraxella catarrhalis* being significantly reduced in the supplemented group (225). Later, the same group published data on the fecal microbiome and metabolome of infant fed formula supplemented with MFGM, standard formula or breast milk, as a reference (226). The effect of MFGM on fecal microbiota was moderate and did not override the effect of formula. Much of this research has used a fairly general MFGM preparation from bovine milk, or even a mixture of MFGM with other compounds (probiotics, prebiotics and lactoferrin) (217, 227, 228) which makes it difficult to isolate the effect of MFGM on the microbiota. The effects of polar lipids, such as phospholipids (229) and gangliosides (230) on the gut microbiota has been demonstrated.

In one *in vivo* study, artificially reared newborn rats supplemented with bovine whey-derived MFGM had similar microbiota to the dam-reared pups compared to non-supplemented pups (78). Another study found that feeding formula with bovine MFGM to piglets decreased the proportions of *Firmicutes* and increased *Proteobacteria* and *Bacteroides* in the gut compared to piglets fed formula with vegetable oils. The effects of undigested MFGM *in vitro* have been demonstrated (231) suggesting that MFGM components may play a role on the infant's gut microbiota development. There is a need for further research to more clearly identify the specific MFGM components that may confer these benefits.

### Metabolic Health

Intake of milk sourced cholesterol in early life was shown to correspond to infants' serum cholesterol levels, which are high in breast-fed compared to formula-fed infants (232). Higher levels of serum cholesterol were shown to prevent cardiovascular diseases in adult life (121, 233) by downregulating hepatic hydroxymethyl glutaryl coenzyme A reductase *via* epigenetic modifications (234).

## Conclusions

**Figure 7:**
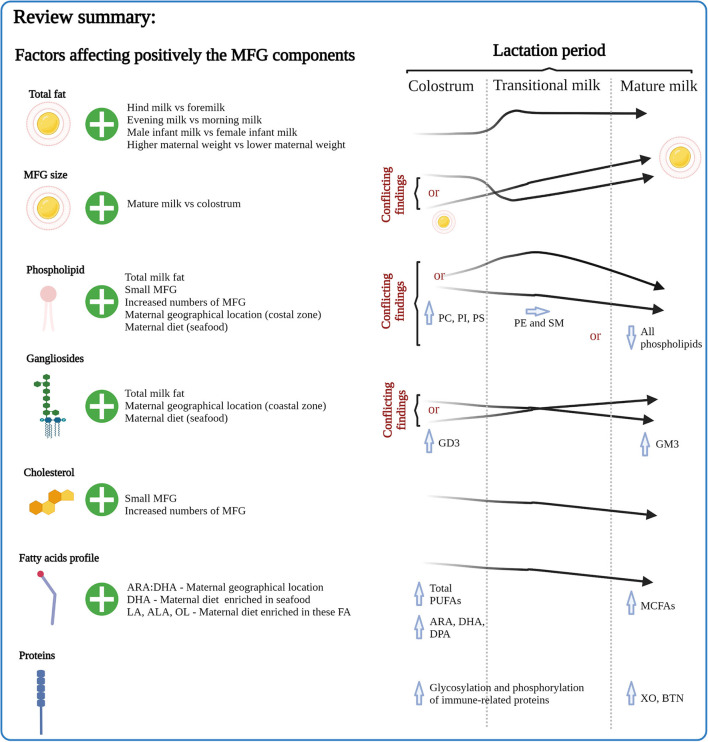
Summary of the factors affecting MFG composition emphasizing the effects of lactation period. MFG, milk fat globule; ARA, arachidonic acid; DHA, docosahexaenoic acid; DPA, docosapentaenoic acid; ALA, α-linolenic acid; LA, linoleic acid; OL, oleic acid; XO, xanthine oxidase; BTN, butyrophilin.

Although the body of literature describing compositional analysis of MFGM components within HM is not standardized in terms of methodology, the results do show certain patterns. For example, the relative concentration levels of MFGM-specific phospholipids, gangliosides, cholesterol, FAs and proteins appear to alter over the course of lactation, and such changes are likely to reflect the changing requirements of the growing infant (review summary, Figure 7). There is also evidence that factors such as maternal diet and geographical location can influence certain aspects of HM MFGM composition.

Although the majority of the research has been conducted using MFGM materials derived from bovine milk and employs animal models, the body of evidence for specific health benefits of MFGM components is increasing, and clearly demonstrates that in addition to its role in encapsulating and delivering lipids within milk, the MFGM also contains a range of components that have important implications for the health of the growing infant. Health outcomes include cognitive development, intestinal development and function, and immune health.

The small number of clinical trials in which infant formula products have been tested provide some evidence that the inclusion of MFGM, or components such as gangliosides, in infant formula has benefits for the health of the growing infant. Although current infant formula products are adequate to support growth and development, comparisons with breast-fed infants suggest that these formula products are not optimal, in particular regarding cognitive outcomes. The data from *in vivo* studies and clinical trials, suggest that the inclusion of MFGM or components within it in infant formula is important to ensure optimal cognitive, immune, and intestinal development and function.

Collectively, the information presented here suggests that infant formula products require further development to mimic the composition of HM more closely, including the MFGM. The production of MFGM ingredients to supplement infant formulations could be tailored to meet the different stages of lactation, or even the different maternal geographical locations. This will ensure that those mothers for whom breast feeding is not possible can have the best possible alternative to their growing infant. To enable this, further work is required to fully understand patterns of MFGM components across lactation, and their efficacy in supporting the health of the infant at different stages of growth and development.

## Author Contributions

CT and MB wrote the manuscript. LD, IS, FL, and YY critically revised the manuscript. All authors have read and agreed to the published version of the manuscript.

## Funding

Authors received funding from Beijing Yili Technology Development Co. and Inner Mongolia Yili Industrial Group, Co., Ltd. The funders were not involved in the study design, collection, analysis, interpretation of data, the writing of this article or the decision to submit it for publication.

## Conflict of Interest

IS, YY, and FL were employed by Inner Mongolia Yili Industrial Group, Co., Ltd, and Inner Mongolia Dairy Technology Research Institute Co., Ltd. CT, LD, and MB were employed by AgResearch Ltd. The remaining author declares that the research was conducted in the absence of any commercial or financial relationships that could be construed as a potential conflict of interest.

## Publisher's Note

All claims expressed in this article are solely those of the authors and do not necessarily represent those of their affiliated organizations, or those of the publisher, the editors and the reviewers. Any product that may be evaluated in this article, or claim that may be made by its manufacturer, is not guaranteed or endorsed by the publisher.

## Abbreviations

HM: Human milk
MFG: milk fat globule
MFGM: milk fat globule membrane
FA: fatty acids
LA: linoleic acid
ALA: α-linolenic acid
EPA: eicosapentaenoic
DHA: docosahexaenoic acid
ARA: arachidonic acid
PUFAs: polyunsaturated fatty acids
MUFA: monounsaturated fatty acids
SFA: saturated fatty acids
MCFA: medium chain fatty acids
DPA: docosapentaenoic acid
OL: oleic acid
XO: xanthine oxidase
BTN: butyrophilin
ADPH: adipophilin
MEC: mammary epithelium cells
MUC: mucin
ER: endoplasmic reticulum
TAG: triacylglycerol
PC: phosphatidylcholine
PE: phosphatidylethanolamine
PS: phosphatidylserine
PI: phosphatidylinositol
SM: sphingomyelin
GD3: disialoganglioside
GM3: monosialodihexosylganglioside.
