# Supplementary material for: Changes in Human Milk Fat Globule Composition Throughout Lactation: A Review
Source: Front Nutr. 2022 May 12;9:835856. doi: 10.3389/fnut.2022.835856 (PMC9137899; doi:10.3389/fnut.2022.835856)
Supplement: Supplementary file 1 [file Data_Sheet_1.PDF]

1 Supplementary Material

Changes in milk fat globule composition throughout lactation: a review

Search strategy

Scopus
((TITLE-ABS-KEY ((milk AND fat AND globule) OR ("milk fat globule" AND membrane)) AND TITLE-ABS-KEY (("breast milk" OR breastmilk* OR "human milk" OR "breast feed" OR breastfeed OR "breast fed" OR breastfed OR lactation)) AND TITLEABS-KEY ("complex lipid" OR fats OR "fatty acid" OR ganglioside* OR phospholipid* OR cholesterol OR (milk AND protein*) OR omega-3 OR omega-6 OR "Arachidonic acid" OR "Docosahexaenoic acid" OR dha OR "Eicosapentaenoic acid" OR epa OR "Linoleic acid")) AND PUBYEAR > 1989) AND NOT (TITLE-ABS-KEY (nonhuman OR rat* OR mouse OR mice OR dairy OR cow* OR bovine OR cattle OR buffalo* OR sheep OR ovine OR pig* OR swine OR rabbit* OR chick* OR goat* OR horse* OR cat* OR dog* OR hamster* OR deer OR calf OR raccoon* OR donkey* OR elephant* OR disease* OR infection* OR cancer* OR carcinoma* OR tumo?r OR pathogen* OR circadian)) AND (LIMITTO (LANGUAGE , "English"))
= 373 results

Medline
exp Lactation/ AND (exp Breast Feeding/ or exp Milk, Human/) AND (exp Milk Proteins/ or exp Fatty Acids/ or exp Nutrients/ or exp Cholesterol/ or exp Glycoproteins/ or exp Glycolipids/ or exp Gangliosides/ or exp Phospholipids/ or exp Fatty Acids, Omega-3/ or exp Fatty Acids, Omega-6/ or exp Eicosapentaenoic Acid/ or exp Arachidonic Acids/ or exp Arachidonic Acid/ or exp Docosahexaenoic Acids/ or exp Linoleic Acids/ or exp Linoleic Acid/) NOT (exp "diseases (non mesh)"/ or disease/ or exp Circadian Rhythm/ or exp Circadian Clocks/ or exp Period Circadian Proteins/ or exp "Circadian Rhythm Signaling Peptides and Proteins"/ or circadian or infection* or cancer* or carcinoma* or tumo?r or pathogen* or non-human or rat* or mouse or mice or dairy or cow* or bovine or cattle or buffalo* or sheep or ovine or pig* or swine or rabbit* or chick* or goat* or horse* or cat* or dog* or hamster* or deer or calf or raccoon* or donkey* or elephant*)
Limit to: English language and Publication Year 1990 – current

=**427 results**
Note: Limits have to applied once the search string is entered

Medline

▼ Search History (8)

# ▲	Searches	Results	Type	Actions	An
1	exp Milk, Human/	20172	Advanced	Display Results | More ▾	
2	exp Breast Feeding/	39454	Advanced	Display Results | More ▾	
3	("milk fat globule" or "milk fat globule membrane").mp. [mp=title, abstract, original title, name of substance word, subject heading word, floating sub-heading word, keyword heading word, organism supplementary concept word, protocol supplementary concept word, rare disease supplementary concept word, unique identifier, synonyms]	1557	Advanced	Display Results | More ▾	
4	1 or 2 or 3	55017	Advanced	Display Results | More ▾	
5	(macronutrient* or nutrient* or "complex lipid" or fats or "fatty acid" or ganglioside* or phospholipid* or cholesterol or "milk fat globule protein" or composition or component*).mp. [mp=title, abstract, original title, name of substance word, subject heading word, floating sub-heading word, keyword heading word, organism supplementary concept word, protocol supplementary concept word, rare disease supplementary concept word, unique identifier, synonyms]	2137261	Advanced	Display Results | More ▾	
6	4 and 5	6967	Advanced	Display Results | More ▾	
7	exp Lactation/	43897	Advanced	Display Results | More ▾	
8	6 and 7	1233	Advanced	Display Results | More ▾	

= 1233 downloaded

Table S 1 Total fat concentration in human colostrum, transitional, and mature milks. The concentration is shown as mean ± standard deviation. Due to some variability in units used, these are specified. NR. Not reported

<table>
<tr><th rowspan="2">Sample collection</th><th rowspan="2">Sampling time</th><th rowspan="2">Units</th><th rowspan="2">Colostrum</th><th rowspan="2">Transitional</th><th colspan="6">Mature milk</th><th rowspan="2">Reference</th></tr>
<tr><th>1 month</th><th>2 months</th><th>3 months</th><th>4 months</th><th>5 months</th><th>6 months</th></tr>
<tr><td>Pooled milk</td><td>24h</td><td>g/100 mL</td><td>2.9 ± 0.3</td><td></td><td></td><td>4.9 ± 0.2</td><td></td><td></td><td></td><td></td><td>(Bauer and Gerss, 2011)</td></tr>
<tr><td>Pooled milk</td><td>24h</td><td>g/L</td><td></td><td></td><td></td><td></td><td>36.2 ± 7.0</td><td></td><td></td><td>37.7 ± 9.6</td><td>(Nommsen et al., 1991)</td></tr>
<tr><td>Single full-breast expression</td><td>NR</td><td>g/100 mL</td><td></td><td></td><td>4.2 ± 0.9</td><td></td><td></td><td>4.6 ± 2.1</td><td></td><td></td><td>(Thakkar et al., 2013)</td></tr>
<tr><td>Single full-breast expression</td><td>NR</td><td>g/L</td><td>28.2 ± 8.6</td><td>35.9 ± 11.6</td><td>34.8 ± 13.8</td><td></td><td></td><td></td><td></td><td></td><td>(Garcia et al., 2011)</td></tr>
<tr><td>Single full-breast expression</td><td>NR</td><td>g/100 mL</td><td></td><td></td><td>3.3 ± 1.2</td><td>3.9 ± 1.2</td><td>3.7 ± 1.0</td><td>3.1 ± 1.3</td><td></td><td>3.5 ± 1.3</td><td>(Ma et al., 2015a)</td></tr>
<tr><td>Single full-breast expression</td><td>Morning</td><td>g/100 mL</td><td>2.9 ± 1.1</td><td>3.6 ± 1.4</td><td colspan="2">3.5 ± 1.6</td><td colspan="2">3.1 ± 1.4</td><td colspan="2">3.2 ± 1.6</td><td>(Yang et al., 2014)</td></tr>
<tr><td>Single full-breast expression</td><td>Morning</td><td>(%)</td><td>3.1 ± 1.0</td><td>4.0 ± 1.7</td><td></td><td></td><td></td><td></td><td></td><td></td><td>(Jiang et al., 2020)</td></tr>
<tr><td>Single full-breast expression</td><td>Morning</td><td>(%)</td><td>3.0 ± 0.4</td><td>3.6 ± 0.5</td><td>4.4 ± 0.6</td><td></td><td></td><td></td><td></td><td></td><td>(Qi et al., 2018)</td></tr>
<tr><td>Pooled foremilk</td><td>24h</td><td>g/100 mL</td><td>2.6 ± 0.8</td><td></td><td>2.0 ± 0.8</td><td>2.3 ± 0.9</td><td>2.1 ± 0.8</td><td>1.6 ± 0.9</td><td>1.1 ± 0.6</td><td>1.9 ± 0.4</td><td rowspan="2">(Saarela et al., 2005)</td></tr>
<tr><td>Pooled hindmilk</td><td>24h</td><td>g/100 mL</td><td>5.9 ± 1.6</td><td></td><td>4.8 ± 1.7</td><td>5.1 ± 1.1</td><td>5.7 ± 1.4</td><td>6.1 ± 1.3</td><td>5.1 ± 2.0</td><td>5.7 ± 2.7</td></tr>
<tr><td>NR</td><td>Morning</td><td>g/100 mL</td><td>1.1 ± 0.3</td><td></td><td>2.9 ± 0.5</td><td></td><td></td><td></td><td></td><td></td><td>(Barbas and Herrera, 1998)</td></tr>
<tr><td>NR</td><td>Morning</td><td>g/L</td><td>37.8 ± 16.5</td><td>51.2 ± 19.5</td><td>53.95 ± 14.6</td><td></td><td></td><td></td><td></td><td></td><td rowspan="2">(Kociszewska-Najman et al., 2012)</td></tr>
<tr><td>NR</td><td>Night</td><td>g/L</td><td>43.2 ± 17.6</td><td>56.6 ± 19.6</td><td>60.7 ± 7.5</td><td></td><td></td><td></td><td></td><td></td></tr>
<tr><td>Foremilk</td><td>Morning</td><td>g/L</td><td>33.3 ± 14.1</td><td>32.5 ± 14.9</td><td>34.0 ± 13.7</td><td></td><td></td><td></td><td></td><td></td><td rowspan="3">(Leghi et al., 2021)</td></tr>
<tr><td>Foremilk</td><td>Afternoon</td><td>g/L</td><td>36.8 ± 11.3</td><td>43.4 ± 20.2</td><td>39.0 ± 15.1</td><td></td><td></td><td></td><td></td><td></td></tr>
<tr><td>Foremilk</td><td>Night</td><td>g/L</td><td>33.2 ± 7.7</td><td>34.4 ± 13.2</td><td>35.8 ± 13.5</td><td></td><td></td><td></td><td></td><td></td></tr>
<tr><td>Foremilk</td><td>NR</td><td>g/L</td><td>nr</td><td></td><td>31.7 ± 16.4</td><td></td><td>28.2 ± 17</td><td></td><td></td><td>26.3 ± 11.1</td><td>(Antonakou et al., 2013)</td></tr>
<tr><td>Foremilk</td><td>NR</td><td>g/100 mL</td><td>1.5 +1 .5</td><td>3.6 +2 .5</td><td>3.3 + 2.6</td><td></td><td></td><td></td><td></td><td></td><td>(Macias and Schweigert, 2001)</td></tr>
<tr><td>Mother <35 years old</td><td rowspan="2">Morning colostrum, otherwise evening</td><td>g/100 mL</td><td>1.8 ± 1.1</td><td>3.5 ± 1.3</td><td>4.6 ± 1.1</td><td></td><td></td><td></td><td></td><td></td><td rowspan="2">(Lubetzky et al., 2015)</td></tr>
<tr><td>Mother > 35 years old</td><td>g/100 mL</td><td>2.7 ± 1.9</td><td>3.9 ± 1.4</td><td>4.1 ± 1.1</td><td></td><td></td><td></td><td></td><td></td></tr>
<tr><td>Mid-breastfeeding</td><td>NR</td><td>g/100 mL</td><td>2.7 ± 1.0</td><td>2.8 ± 1.1</td><td colspan="3">3.7 ± 1.4</td><td colspan="3">3.2 ± 1.5</td><td>(Yamawaki et al., 2005)</td></tr>
<tr><td>Single full-breast expression</td><td>NR</td><td>g/100 mL</td><td>2.7 ± 1.0</td><td>3.0 ± 0.9</td><td>3.1 ± 1.5</td><td>3.2 ± 1.3</td><td>3.0 ± 1.4</td><td>2.9 ± 1.4</td><td>3.0 ± 1.4</td><td>3.1 ± 1.4</td><td>(Chang et al., 2015)</td></tr>
<tr><td>NR</td><td>NR</td><td>g/100 mL</td><td>2.3 ± 0.0</td><td>3.3 ± 0.0</td><td colspan="2">3.2 ± 0.2</td><td colspan="2">3.3 ± 0.1</td><td colspan="2">2.4 ± 0.1</td><td rowspan="3">(Claumarchirant et al., 2016)</td></tr>
<tr><td>NR</td><td>NR</td><td>g/100 mL</td><td rowspan="2">2.3 ± 0.1</td><td rowspan="2">4.9 ± 0.0</td><td colspan="2">4.0 ± 0.0</td><td colspan="2">3.2 ± 0.1</td><td colspan="2">3.3 ± 0.0</td></tr>
<tr><td>NR</td><td>NR</td><td>g/100 mL</td><td colspan="2">3.5 ± 0.1</td><td colspan="2">3.1 ± 0.1</td><td colspan="2">2.7 ± 0.1</td></tr>
</table>

Table S 2 Methodology and number of mothers sampled for the analysis of total milk fat, phospholipids and gangliosides.

Reference	Parameter measured	Number of mothers	Method Extraction	Sample collection	Extraction time	Sample preservation	Method of analysis
(Thakkar et al., 2013)	Phospholipids	50	Mechanical	Single full-breast expression	NR	Frozen -80°C	HPLC- ELSD
(Giuffrida et al., 2016)	Phospholipids	113	Mechanical	Single full-breast expression	Morning	Frozen -80°C	HPLC- ELSD
(Giuffrida et al., 2016)	Phospholipids	112	Mechanical	Single full-breast expression	Morning	Frozen -80°C	HPLC- ELSD
(Giuffrida et al., 2016)	Phospholipids	120	Mechanical	Single full-breast expression	Morning	Frozen -80°C	HPLC- ELSD
(Jiang et al., 2018)	Phospholipids	18	Mechanical	Single full-breast expression	NR	Frozen -80°C	HPLC-IT TOF-MS
(Wu et al., 2019)	Phospholipids	168	Mechanical	Double full-breast expression	Morning	Frozen -80°C	HPLC-VWD
(Wei et al., 2019)	Phospholipids	6	Mechanical	Single full-breast expression	Morning	Frozen -20°C (<2h), then frozen -80°C	^{31}P NMR spectra
(Ingvordsen Lindahl et al., 2019)	Phospholipids	22	NR	ND sample from milk bank	NR	Frozen -80°C	LC/ESI-MS
(Garcia et al., 2011)	Phospholipids	22	Mechanical	Single full-breast expression	NR	Frozen −20°C	TLC, GC-FAME
(Zou et al., 2012)	Phospholipids	45	NR	NR	NR	Frozen −20°C	HPLC- ELSD
(Sala-Vila et al., 2005)	Phospholipids	30	Mechanical	Double full-breast expression Subsamples – pooled foremilk	NR	Frozen -80°C	HPLC- ELSD
(Claumarchirant et al., 2016)	Phospholipids	(20-22)	NR	pooled	NR	Frozen −20°C	HPLC- ELSD
	Phospholipids	(8-22)	NR	pooled	NR	Frozen −20°C	HPLC- ELSD
	Phospholipids		NR	pooled	NR	Frozen −20°C	HPLC- ELSD
(McJarrow et al., 2019)	Phospholipids	41	NR	NR	NR	NR	HPLC–MS
(Ma et al., 2017)	Phospholipids	26	Mechanical	Single full-breast expression	NR	Frozen -80°C	HPLC–MS
(Rueda et al., 1995)	Gangliosides	18	Mechanical	Foremilk expressed	Morning	Frozen after collection	HPTLC
(Rueda et al., 1996)	Gangliosides	51 (Spain) 18 (Panama)	Mechanical	Foremilk expressed from the breast	Morning	Frozen after collection	HPTLC
(Pan and Izumi, 1999)	Gangliosides	19	NR	NR	NR	Frozen at -20°C after collection	HPTLC
(Martin-Sosa et al., 2004)	Gangliosides	12	Mechanical or manual	NR	NR	Stored at −20°C, lyophilized, and homogenized	HPTLC
(Ma et al., 2015b)	Gangliosides	48	Mechanical	Single full-breast expression	NR	Frozen -80°C	HPLC-MS

(Ma et al., 2015a)	Gangliosides	20 samples at each time point	Mechanical	Single full-breast expression	NR	Frozen -80°C	HPLC-MS
	Gangliosides	14 samples at 1 month	Mechanical	Single full-breast expression	NR	Frozen -80°C	HPLC-MS
(Tan et al., 2020)	Gangliosides	47	NR	NR	NR	Frozen -80°C	HPLC-MS
(Giuffrida et al., 2014)	Gangliosides	450	Mechanical	Single full-breast expression	NR	Frozen -80°C	HPLC-MS
(Giuffrida et al., 2016)	Gangliosides	540	Mechanical	Single full-breast expression	Morning	Frozen -80°C	HPLC-MS
(Thakkar et al., 2013)	Gangliosides	50	Mechanical	Single full-breast expression	NR	Frozen -80°C	HPLC-MS
(McJarrow et al., 2019)	Gangliosides	41	NR	NR	NR	NR	HPLC-MS
(Bauer and Gerss, 2011)	Total Fat	10	Mechanical	Pooled milk	24h	Frozen -70°C	Creamatocrit
(Nommsen et al., 1991)	Total Fat	92	Mechanical	Pooled milk, single full-breast expression	24h	NR	Folch extraction
(Thakkar et al., 2013)	Total Fat	50	Mechanical	Single full-breast expression	NR	Frozen -80°C	MIRIS human milk analyzer
(Garcia et al., 2011)	Total Fat	22	Mechanical	Single full-breast expression	NR	Frozen −20°C	TLC
(Ma et al., 2015a)	Total Fat	34	Mechanical	Single full-breast expression	NR	Frozen -80°C	MIRIS human milk analyzer
(Yang et al., 2014)	Total Fat	436	Mechanical or manual	Single full-breast expression	Morning	Frozen -80°C	MIRIS human milk analyzer
(Jiang et al., 2020)	Total Fat	6	Mechanical	Single full-breast expression	Morning	Frozen -80°C	Rose Gottlieb
(Qi et al., 2018)	Total Fat	103	NR	NR	Morning	Frozen -80°C	Mojonnier method
(Saarela et al., 2005)	Total Fat	53	Mechanical or manual	Pooled foremilk	24h	Frozen −20°C	Rose Gottlieb
	Total Fat	53	Mechanical or manual	Pooled hindmilk	24h	Frozen -20°C	Rose Gottlieb
(Barbas and Herrera, 1998)	Total Fat	15	Mechanical	NR	Morning	Frozen -80°C	Creamatocrit
(Kociszewska-Najman et al., 2012)	Total Fat	39	NR	NR	Morning and night	Stored at room temperature	Creamatocrit
(Leghi et al., 2021)	Total Fat	15	Mechanical or manual	Double full-breast expression	Morning	Frozen -80°C	Creamatocrit
	Total Fat		Mechanical or manual	Double full-breast expression	Afternoon	Frozen -80°C	Creamatocrit
	Total Fat		Mechanical or manual	Double full-breast expression	Night	Frozen -80°C	Creamatocrit
(Antonakou et al., 2013)	Total Fat	39	Mechanical	Foremilk, single breast	Morning	Frozen -80°C	Creamatocrit
(Macias and Schweigert, 2001)	Total Fat	21	Manual	Foremilk, single breast	Morning	Frozen -80°C	Creamatocrit
(Lubetzky et al., 2015)	Total Fat	34	Manual	Mother younger than 35 years old	Morning colostrum,	Frozen -80°C	MIRIS human milk analyzer

	Total Fat	38	Manual	Mother older than 35 years old, Morning colostrum, evening transitional and mature milk	evening transitional and mature milk		
(Yamawaki et al., 2005)	Total Fat	4000	NR	Mid-breastfeeding	NR	Frozen -40°C	Rose Gottlieb method
(Chang et al., 2015)	Total Fat	2632	Mechanical	Single full-breast expression	No specific time	Frozen −20°C	MilkoScan FT2
(Claumarchirant et al., 2016)	Total Fat	20-22	NR	Pooled milk	NR	Frozen −20°C	Rose Gottlieb method
		8-22	NR	Pooled milk	NR	Frozen −20°C	Rose Gottlieb method

NR. Not reported. GC-FAME, Gas Chromatographic Analysis for Fatty Acid Methyl Ester; TLC, Thin-layer chromatography; HPTLC, High-performance thin-layer chromatography; HPLC, High performance liquid chromatography; ELSD, evaporative light scattering detector; MS, Mass spectrometry; VWD, Variable Wavelength Ultraviolet Detector; LC/ESI-MS, Liquid Chromatography Electrospray Ionization Tandem Mass Spectrometric; ^{31}P NMR spectra, Phosphorus-31 nuclear magnetic resonance.

References

Antonakou, A., Skenderi, K.P., Chiou, A., Anastasiou, C.A., Bakoula, C., and Matalas, A.L. (2013). Breast milk fat concentration and fatty acid pattern during the first six months in exclusively breastfeeding Greek women. *European Journal of Nutrition* 52(3)**,** 963-973. doi: https://dx.doi.org/10.1007/s00394-012-0403-8.

Barbas, C., and Herrera, E. (1998). Lipid composition and vitamin E content in human colostrum and mature milk. *J Physiol Biochem* 54(3)**,** 167-173.

Bauer, J., and Gerss, J. (2011). Longitudinal analysis of macronutrients and minerals in human milk produced by mothers of preterm infants. *Clinical Nutrition* 30(2)**,** 215-220. doi: https://dx.doi.org/10.1016/j.clnu.2010.08.003.

Chang, N., Jung, J.A., Kim, H., Jo, A., Kang, S., Lee, S.-W., et al. (2015). Macronutrient composition of human milk from Korean mothers of full term infants born at 37-42 gestational weeks. *Nutrition research and practice* 9(4)**,** 433-438.

Claumarchirant, L., Cilla, A., Matencio, E., Sanchez-Siles, L.M., Castro-Gomez, P., Fontecha, J., et al. (2016). Addition of milk fat globule membrane as an ingredient of infant formulas for resembling the polar lipids of human milk. *International Dairy Journal* 61**,** 228-238. doi: 10.1016/j.idairyj.2016.06.005.

Garcia, C., Millet, V., Coste, T.C., Mimoun, M., Ridet, A., Antona, C., et al. (2011). French mothers' milk deficient in DHA contains phospholipid species of potential interest for infant development. *J Pediatr Gastroenterol Nutr* 53(2)**,** 206-212. doi: 10.1097/MPG.0b013e318216f1d0.

Giuffrida, F., Cruz-Hernandez, C., Bertschy, E., Fontannaz, P., Masserey Elmelegy, I., Tavazzi, I., et al. (2016). Temporal Changes of Human Breast Milk Lipids of Chinese Mothers. *Nutrients* 8(11)**,** 10.

Giuffrida, F., Elmelegy, I.M., Thakkar, S.K., Marmet, C., and Destaillats, F. (2014). Longitudinal evolution of the concentration of gangliosides GM3 and GD3 in human milk. *Lipids* 49(10)**,** 997-1004. doi: 10.1007/s11745-014-3943-2.

Ingvordsen Lindahl, I.E., Artegoitia, V.M., Downey, E., O'Mahony, J.A., O'Shea, C.A., Ryan, C.A., et al. (2019). Quantification of Human Milk Phospholipids: the Effect of Gestational and Lactational Age on Phospholipid Composition. *Nutrients* 11(2)**,** 22. doi: https://dx.doi.org/10.3390/nu11020222.

Jiang, C., Ma, B., Song, S., Lai, O.M., and Cheong, L.Z. (2018). Fingerprinting of Phospholipid Molecular Species from Human Milk and Infant Formula Using HILIC-ESI-IT-TOF-MS and Discriminatory Analysis by Principal Component Analysis. *Journal of Agricultural and Food Chemistry* 66(27)**,** 7131-7138. doi: 10.1021/acs.jafc.8b01393.

Jiang, W., Zhang, X., Cheng, J., Song, J., Jin, Q., Wei, W., et al. (2020). Variation of fat globule size and fatty acids in human milk in the first 30 days of lactation. *International Dairy Journal* 100. doi: 10.1016/j.idairyj.2019.104567.

Kociszewska-Najman, B., Borek-Dzieciol, B., Szpotanska-Sikorska, M., Wilkos, E., Pietrzak, B., and Wielgos, M. (2012). The creamatocrit, fat and energy concentration in human milk produced by mothers of preterm and term infants. *Journal of Maternal-Fetal & Neonatal Medicine* 25(9)**,** 1599-1602. doi: https://dx.doi.org/10.3109/14767058.2011.648239.

Leghi, G.E., Lai, C.T., Narayanan, A., Netting, M.J., Dymock, M., Rea, A., et al. (2021). Daily variation of macronutrient concentrations in mature human milk over 3 weeks. *Scientific Reports* 11(1). doi: 10.1038/s41598-021-89460-5.

Lubetzky, R., Sever, O., Mimouni, F.B., and Mandel, D. (2015). Human Milk Macronutrients Content: Effect of Advanced Maternal Age. *Breastfeeding Medicine: The Official Journal of the Academy of Breastfeeding Medicine* 10(9)**,** 433-436. doi: https://dx.doi.org/10.1089/bfm.2015.0072.

Ma, L., Liu, X., MacGibbon, A.K., Rowan, A., McJarrow, P., and Fong, B.Y. (2015a). Lactational changes in concentration and distribution of ganglioside molecular species in human breast milk from Chinese mothers. *Lipids* 50(11)**,** 1145-1154. doi: https://dx.doi.org/10.1007/s11745-015-4073-1.

Ma, L., MacGibbon, A.K., Mohamed, H.J.B.J., Loy, S., Rowan, A., McJarrow, P., et al. (2017). Determination of phospholipid concentrations in breast milk and serum using a high performance liquid chromatography–mass spectrometry–multiple reaction monitoring method. *International Dairy Journal* 71**,** 50-59.

Ma, L., MacGibbon, A.K.H., Jan Mohamed, H.J.B., Loy, S., Rowan, A., McJarrow, P., et al. (2015b). Determination of ganglioside concentrations in breast milk and serum from Malaysian mothers using a high performance liquid chromatography-mass spectrometry-multiple reaction monitoring method. *International Dairy Journal* 49**,** 62-71. doi: 10.1016/j.idairyj.2015.05.006.

Macias, C., and Schweigert, F.J. (2001). Changes in the concentration of carotenoids, vitamin A, alpha-tocopherol and total lipids in human milk throughout early lactation. *Annals of Nutrition & Metabolism* 45(2)**,** 82-85.

Martin-Sosa, S., Martin, M.J., Castro, M.D., Cabezas, J.A., and Hueso, P. (2004). Lactational changes in the fatty acid composition of human milk gangliosides. *Lipids* 39(2)**,** 111-116.

McJarrow, P., Radwan, H., Ma, L., MacGibbon, A.K.H., Hashim, M., Hasan, H., et al. (2019). Human Milk Oligosaccharide, Phospholipid, and Ganglioside Concentrations in Breast Milk from United Arab Emirates Mothers: Results from the MISC Cohort. *Nutrients* 11(10)**,** 08. doi: https://dx.doi.org/10.3390/nu11102400.

Nommsen, L.A., Lovelady, C.A., Heinig, M.J., Lonnerdal, B., and Dewey, K.G. (1991). Determinants of energy, protein, lipid, and lactose concentrations in human milk during the first 12 mo of lactation: the DARLING Study. *American Journal of Clinical Nutrition* 53(2)**,** 457-465.

Pan, X.L., and Izumi, T. (1999). Chronological changes in the ganglioside composition of human milk during lactation. *Early Human Development* 55(1)**,** 1-8.

Qi, C., Sun, J., Xia, Y., Yu, R., Wei, W., Xiang, J., et al. (2018). Fatty Acid Profile and the sn-2 Position Distribution in Triacylglycerols of Breast Milk during Different Lactation Stages. *Journal of Agricultural & Food Chemistry* 66(12)**,** 3118-3126. doi: https://dx.doi.org/10.1021/acs.jafc.8b01085.

Rueda, R., Maldonado, J., and Gil, A. (1996). Comparison of content and distribution of human milk gangliosides from Spanish and Panamanian mothers. *Annals of Nutrition & Metabolism* 40(4)**,** 194-201.

Rueda, R., Puente, R., Hueso, P., Maldonado, J., and Gil, A. (1995). New data on content and distribution of gangliosides in human milk. *Biological Chemistry Hoppe-Seyler* 376(12)**,** 723-727.

Saarela, T., Kokkonen, J., and Koivisto, M. (2005). Macronutrient and energy contents of human milk fractions during the first six months of lactation. *Acta Paediatrica* 94(9)**,** 1176-1181.

Sala-Vila, A., Castellote, A.I., Rodriguez-Palmero, M., Campoy, C., and Lopez-Sabater, M.C. (2005). Lipid composition in human breast milk from Granada (Spain): changes during lactation. *Nutrition* 21(4)**,** 467-473.

Tan, S., Chen, C., Zhao, A., Wang, M., Zhao, W., Zhang, J., et al. (2020). The dynamic changes of gangliosides in breast milk and the intake of gangliosides in maternal and infant diet in three cities of China. *International Journal of Clinical and Experimental Pathology* 13(11)**,** 2870.

Thakkar, S.K., Giuffrida, F., Cristina, C.H., De Castro, C.A., Mukherjee, R., Tran, L.A., et al. (2013). Dynamics of human milk nutrient composition of women from Singapore with a special focus on lipids. *American Journal of Human Biology* 25(6)**,** 770-779. doi: https://dx.doi.org/10.1002/ajhb.22446.

Wei, W., Yang, J., Yang, D., Wang, X., Yang, Z., Jin, Q., et al. (2019). Phospholipid Composition and Fat Globule Structure I: Comparison of Human Milk Fat from Different Gestational Ages, Lactation Stages, and Infant Formulas. *Journal of Agricultural & Food Chemistry* 67(50)**,** 13922-13928. doi: https://dx.doi.org/10.1021/acs.jafc.9b04247.

Wu, K., Gao, R., Tian, F., Mao, Y., Wang, B., Zhou, L., et al. (2019). Fatty acid positional distribution (sn-2 fatty acids) and phospholipid composition in Chinese breast milk from colostrum to mature stage. *British Journal of Nutrition* 121(1)**,** 65-73. doi: https://dx.doi.org/10.1017/S0007114518002994.

Yamawaki, N., Yamada, M., Kan-no, T., Kojima, T., Kaneko, T., and Yonekubo, A. (2005). Macronutrient, mineral and trace element composition of breast milk from Japanese women. *Journal of Trace Elements in Medicine & Biology* 19(2-3)**,** 171-181.

Yang, T., Zhang, Y., Ning, Y., You, L., Ma, D., Zheng, Y., et al. (2014). Breast milk macronutrient composition and the associated factors in urban Chinese mothers. *Chinese Medical Journal* 127(9)**,** 1721-1725.

Zou, X.Q., Guo, Z., Huang, J.H., Jin, Q.Z., Cheong, L.Z., Wang, X.G., et al. (2012). Human milk fat globules from different stages of lactation: a lipid composition analysis and microstructure characterization. *Journal of Agricultural & Food Chemistry* 60(29)**,** 7158-7167. doi: https://dx.doi.org/10.1021/jf3013597.
